# Yeast cell factories for fine chemical and API production

**DOI:** 10.1186/1475-2859-7-25

**Published:** 2008-08-07

**Authors:** Beate Pscheidt, Anton Glieder

**Affiliations:** 1 Research Centre Applied Biocatalysis GmbH, Petersgasse 14/3, 8010 Graz, Austria; 2 Institute of Molecular Biotechnology, Graz University of Technology, Petersgasse 14/2, 8010 Graz, Austria

## Abstract

This review gives an overview of different yeast strains and enzyme classes involved in yeast whole-cell biotransformations. A focus was put on the synthesis of compounds for fine chemical and API (= active pharmaceutical ingredient) production employing single or only few-step enzymatic reactions. Accounting for recent success stories in metabolic engineering, the construction and use of synthetic pathways was also highlighted. Examples from academia and industry and advances in the field of designed yeast strain construction demonstrate the broad significance of yeast whole-cell applications. In addition to *Saccharomyces cerevisiae*, alternative yeast whole-cell biocatalysts are discussed such as *Candida *sp., *Cryptococcu*s sp., *Geotrichum *sp., *Issatchenkia *sp., *Kloeckera *sp., *Kluyveromyces *sp., *Pichia *sp. (including *Hansenula polymorpha *= *P. angusta*), *Rhodotorula *sp., *Rhodosporidium *sp., alternative *Saccharomyces *sp., *Schizosaccharomyces pombe*, *Torulopsis *sp., *Trichosporon *sp., *Trigonopsis variabilis*, *Yarrowia lipolytica *and *Zygosaccharomyces rouxii*.

## Background

The advent of yeast whole-cell biocatalysis coincided with the development of first technologies for the human society. Some thousand years BC, when organized agriculture had appeared, the development of yeast-based technology started. Today, artistic records (e.g. in tombs of wealthier members of the ancient Egyptian society) and archaeological data provide an insight into the daily life in ancient Egypt [1,2] and teach us that bread and also beer were central parts of the Egyptian's diet [3]. However, no more than approximately 200 years ago, leading scientists recognized yeasts as the cause of fermentation and started to examine them because of their economic importance and one morphologic advantage compared to some other microorganisms, their large cells [4]. Understanding the scientific basis for alcoholic fermentation was financially well supported by the alcoholic fermentation industries and governments at that time. Basically, research on yeasts greatly contributed to the development of microbiology, biochemistry and also biocatalysis. Berthelot's and Emil Fischer's research results on the utilization of different sugars by yeasts were central to studies on enzymes and their specificity. Especially Fischer's lock and key model published in 1894 [5] provided the basis for subsequent concepts. These include for example the theory about the enzyme-substrate complex developed by Henri [6] and Michaelis & Menten [7], respectively, Haldane's concept of substrate activation representing the idea of selective binding energy which leads either to transition state stabilization or substrate destabilization [8], and the induced fit theory described by Koshland [9,10]. Early yeast research, which was comprehensively reviewed by Barnett [4], also contributed to basic biochemical knowledge including the understanding of metabolic pathways, Monod's concepts of enzyme induction[11] and basic findings on cell cycles [12,13].

Most of this early research was based on *Saccharomyces cerevisiae *and therefore, the term 'yeast' was and is often taken as a synonym for *S. cerevisiae*. However, yeasts belong to a group of eukaryotic microorganisms, predominantly unicellular and phylogenetically quite diverse [14]. They are assigned to two taxonomic classes of fungi, the ascomycetes and the basidiomycetes [14,15]. Their classification is mostly based on phenotypic characters such as morphology, the ability to utilize various exogenous compounds and modes of vegetative reproduction, namely budding or fission. Some yeasts also form sexual states which differ from those of other fungi as they are not enclosed in fruiting bodies [15]. At the end of the 20^th ^century molecular methods have become increasingly popular in order to estimate genetic relation among yeasts. Recently, Hibbett et al. [16] attempted to create a consensus higher-level classification for the *Fungi *for general use. This broad-based consensus classification was necessary in order to prevent confusion and loss of information as caused by repetitive renaming of several yeast strains in the past [17]. An overview of yeast genera was provided by Walker [18] or Boekhout and Kurtzman [15,19], for example. A comprehensive phylogenetic relationship among sequenced fungal genomes, including more than 20 yeast genomes, was recently depicted by Scannell et al. [20].

Although yeasts, especially *Saccharomyces cerevisiae*, have been employed in synthetic organic chemistry since the beginning of the 20^th ^century [21-23], scientists devoted to classical organic chemistry often hesitated to consider biological systems for their synthetic problems [24]. In the 1970s, biocatalysis started booming and up to now the number of publications on biotransformations has been rising exponentially. Remarkable findings led to a better understanding of biological systems and consequently to their increased application for chemical conversions, especially in the field of organic synthesis [25].

In general, two major synthetic technologies based on biocatalytic reactions were described, namely 'fermentation' and 'enzymation' (alternative names for 'enzymation': 'microbial transformation', 'microbial conversion', 'biotransformation', 'bioconversion') [24,25]. Fermentation was considered to be a biological method resulting in products which are the result of the complex metabolism of microorganisms starting with inexpensive simple carbon and nitrogen sources. As a consequence, living or even growing cells were a prerequisite for this technology and fermentation was regarded to always result in natural products [25]. On the other hand, enzymation was specified not to necessarily require living cells, as cells were only important for the enzyme's production and were themselves regarded as 'simple bag of enzymes or catalysts' [25]. Furthermore, enzymation was described to be a one or few-step conversion of a more complex substrate into a product [24,25]. However, Yamada and Shimizu [25] already stated that it was not always possible to clearly distinguish between the two categories.

In recent years, the way for the generation of designed microorganisms was paved by an increasing number of sequenced genes and even whole genomes, new bioinformatic tools providing the basis for analyzing this wealth of information, biochemically well-characterized biosynthetic pathways and well-established and facile genetic engineering techniques. These approaches include for example, the construction of synthetic pathways for the production of structurally complex, natural products like isoprenoids or polyketides and novel variations thereof. Furthermore, even minimum genome factories could enter the field of biocatalysis in future. Currently, first examples of such cell factories are created in which unnecessary or harmful genes are deleted and only genes necessary for industrial production are present [26]. For this approach, up to now, three species were selected, namely two bacteria (*Escherichia coli *[27] and *Bacillus subtilis *[28]), and the fission yeast *Schizosaccharomyces pombe *[29]. With these developments in mind, one has to admit that the idea of whole-cell biocatalysts being 'black boxes' already started to fade.

The following chapters will now give an overview of different yeast strains and enzyme classes involved in yeast whole-cell biocatalysis. We will provide examples from academia and industry and especially focus on recent advances in the field of designed yeast strains for whole-cell biocatalytic applications. Thereby, we will also include synthetic pathways to structurally complex compounds, a methodology which lies in between classical 'fermentation' and 'enzymation'. Thus, we will include both, one- and multi-step enzymatic reactions in a native or engineered environment, starting with simple or complex substrate molecules.

## Review

### 1. Chemical reactions catalyzed by wild-type yeast whole-cell biocatalysts

Especially baker's yeast (= *Saccharomyces cerevisiae*) was regarded to be ideal for chemists looking for a stereoselective biocatalyst, which should eventually lead to chiral intermediates in the synthesis of enantiomerically pure compounds [30]. It is nonpathogenic, inexpensive, simple to grow at laboratory and large scale and the cells can be stored indefinitely in dried form [24]. For chemical synthesis, however, the chemical repertoire of yeast whole-cell biocatalysts is of major importance. In the following, an overview is given, outlining the main enzymatic reactions performed by wild-type yeast strains, and positive and negative aspects of these whole-cell biocatalysts are discussed.

#### 1.1 Reduction of C=O-bonds

**Figure 1:**
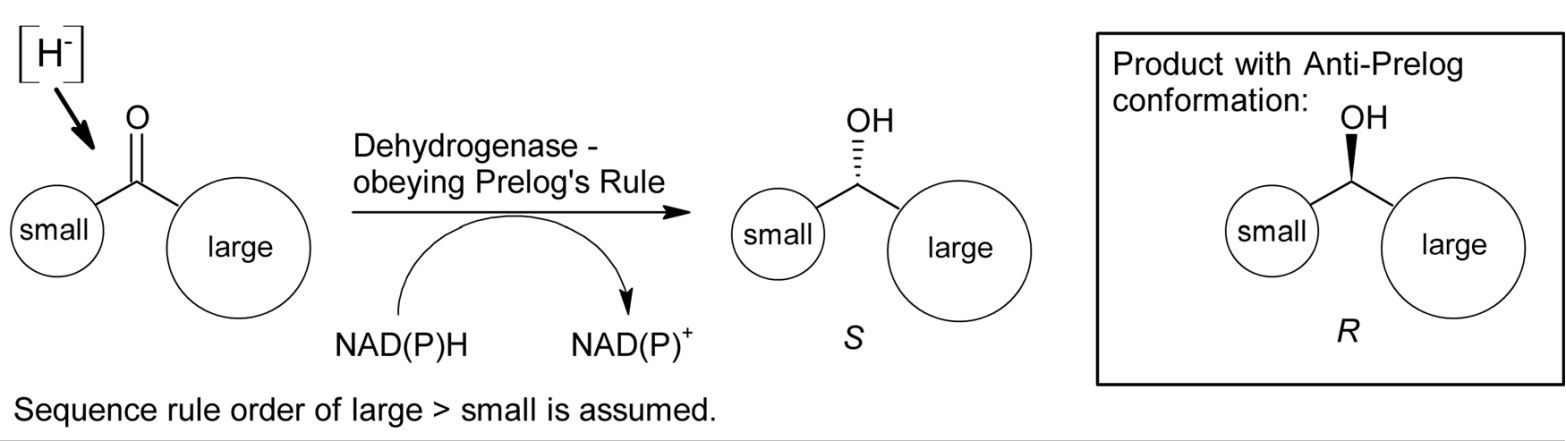
Asymmetric reduction of ketones according to Prelog's Rule [44].

The asymmetric reduction of carbonyl-containing compounds by yeast, in particular *Saccharomyces cerevisiae*, depicts probably the most thoroughly investigated class of whole-cell biotransformations. One of the first reports on this topic was the reduction of furfural to furfuryl alcohol by Windisch [31] and Lintner [32] at the beginning of the 20^th ^century. The first comprehensive overview of reduction reactions catalyzed by yeast was published in 1949 [33]. Since that time, many different substrates containing carbonyl moieties were subjected to yeast bioreduction and the most important achievements were summarized in reviews and book chapters, partly focusing on *Saccharomyces cerevisiae *[30,34,35] but also on biocatalysts in general, including alternative yeasts [24,25,36-43]. The investigated substrate spectrum is huge, including a variety of functional groups as substituents of the ketone moiety (for example heterocyclic-, hydroxyl-, sulfur-, cyano-, and azido-groups, or different halogenides) and even derivatives such as silyl- or germyl-groups were found to be accepted [24]. Generally, *Saccharomyces cerevisiae *reduces simple aliphatic and aromatic ketones according to Prelog's rule [44] resulting in the corresponding (*S*)-alcohols [45] (Figure 1). However, this should not always be generalized and caution should be exercised in particular, when Prelog's rule is applied to whole cells [34].

A major advantage of whole-cell (redox)-biocatalysts is the availability of all the necessary cofactors and metabolic pathways for their regeneration. Furthermore, cheap carbon sources (e.g. glucose, saccharose) can be employed as auxiliary substrates. Finally, the actual biocatalyst and cofactors are well protected within their natural cellular environment which makes the catalytic system more stable [24]. However, employing wild-type yeast strains as whole-cell biocatalysts also includes distinct drawbacks: Most of the interesting substrates are non-natural and toxic to living organisms. Therefore, they must be used in diluted systems at low concentrations (mostly ≤ 0.3% per volume) [24,46]. Only small fractions of the auxiliary substrate are used for cofactor recycling, the majority is metabolized. This results in large amounts of biomass and by-products which impede product recovery tremendously, especially if the product is not secreted to the reaction medium. Transport phenomena into and out of the cell could be encountered which may even influence specificity [24,46]. Eventually, comparable results are only possible if exactly the same culture is used for repetitive biotransformations since different strains of the same microorganism could have different specificities [24,46]. Another major limitation of wild-type yeast strains is the presence of a large number of different dehydrogenases with overlapping substrate specificities but opposite stereoselectivities [47]. The elucidation of the complete genome sequence of *Saccharomyces cerevisiae *[48,49] finally made scientists aware of the huge variety of available oxidoreductases. Earlier, empirical findings helped to develop methods to improve the selectivity of yeast whole-cell biotransformations. These techniques included substrate modification [50], changes in cultivation conditions or the application of different carbon sources [51], the use of inhibitors [52] or the use of two-phase systems [53,54]. Recently, also water immiscible ionic liquids were used as biocompatible solvents for yeast whole-cell biocatalysis in order to provide a substrate reservoir and an *in situ *extracting agent to increase chemical yields [55].

If these techniques were not successful, alternative microorganisms had to be screened. In addition to *Saccharomyces cerevisiae *also alternative yeasts were found which provided valuable biocatalysts for asymmetric carbonyl reduction. Some were efficient enough to be employed for industrial applications by different companies (Table 2 and Additional File 2, Section 2).

Some recent advances in the biocatalytic reduction of carbonyl-containing compounds are summarized in Table 1 (Additional file 1) and include for example the finding of new activities for *Yarrowia lipolytica *[56]. Lagos et al. [56] screened different yeast strains for the enantioselective production of a halohydrin precursor for (*S*)-propranolol synthesis. *Yarrowia lipolytica *1240 (Spanish type culture collection CECT, Valencia) resting cells gave the 'anti-Prelog'-enantiomer (*S*)-1-chloro-3-(1-naphthyloxy)propan-2-ol with 87% yield and 99% ee. In addition, *Pichia mexicana *11015 (CECT, Valencia) resting cells were found to give 85% yield and 95% ee for the (*R*)-enantiomer. Dehli and Gotor [57] discovered that resting cells of *Saccharomyces montanus *CBS 6772 performed the bioreduction of 2-oxo cyclopentane carbonitriles to the corresponding *cis*-hydroxynitriles in 93% ee, and high de and chemical yields.

**Table 1:** Examples of wild-type yeast whole-cell biocatalysts for the reduction of C=O bonds and comparison to *S. cerevisiae*, if provided.

**Whole-Cell Biocatalyst**	**Substrate**	**Product**	**Performance: yield (ee)**	**Ref.**
*Candida parapsilosis *IFO 1396			*C.p*.: 60% (98% *S*)	[61]
			
*Candida arborea *IAM 4147			*C.a*.: 37% (99% *R*)	
*Issatchenkia scutulata *IFO 10070			*I.s*.: 48% (99% *R*)	
*Kluyveromyces lactis *IFO 1267			*K.l*.: 99% (93% *R*)	
			
*Candida tropicalis *PBR-2 MTCC 5158			*C.t*.: > 84% conv.^a ^(> 99% *S*)	[62]
			
*Geotrichum candidum *CBS 233.76 with Amberlite XAD-1180			*G.c*.: 95% (> 98% *S*)	[60]
			
*Rhodotorula mucillaginosa *CBS 2378			*R.m*.: 88% (> 99% *R*)	
*Saccharomyces cerevisiae*^b^			*S.c*.: 95% (41% *R*)	

*Saccharomyces montanus *CBS 6772			*S.m*.: ee_cis _= 93% (1*S*,2*S*) cis:trans = 96:4	[57]
*Saccharomyces cerevisiae *(Type II from Sigma)			*S.c*.: ee_cis _= 90% (1*S*,2*S*) cis:trans = 95:5	

*Saccharomyces cerevisiae *(dried baker's yeast)			*S.c*.: – (94% *R*)	[58]

*Saccharomyces cerevisiae*			*S.c*.: 75% (> 99%); 96% de	[59]

*Pichia mexicana *CECT^c ^11015			*P.m*.: 85–86% (95% *R*)	[56]
*Saccharomyces cerevisiae *CECT^c ^1317			*S.c*.: 48% (75% *R*)	
			
*Yarrowia lipolytica *CECT^c ^1240			*Y.l*.: 87–88% (99% *S*)	
*Saccharomyces cerevisiae *(Type II, Sigma)			*S.c*.: 32% (83% *S*)	

*Kluyveromyces marxianus *CBS 600			*K.m.: ***2-PE: **26.5 g L^-1 ^in org. phase^e ^STY:^f ^0.33 g L^-1 ^h^-1^	[63]
			**2-PEAc: **6.1 g L^-1 ^in org. phase^e ^**STY:**^f ^0.08 g L^-1 ^h^-1^	
*Saccharomyces cerevisiae *GIV 2009^d^			*S.c.: ***2-PE: **24.0 g L^-1 ^in org. phase^g ^after 166 h **STY:**^f ^0.14 g L^-1 ^h^-1^	[64]

^a^conv. = conversion; ^b^baker's yeast from Distillerie Italiane, Eridania group; ^c^Spanish type culture collection CECT, Valencia; ^d^The wild type strain *S. cerevisiae *Giv 2009 was from Givaudan Ltd. (Dübendorf, Switzerland); ^e^organic phase = Polypropylene glycol 1200 [63]; ^f^STY = space-time yield; calculated from *t *= 0 until *t *where the maximum product concentration was reached; ^g^organic phase = oleic acid [64].

As cheap alternative, *Saccharomyces cerevisiae *is still quite often used for laboratory-scale bioreductions. Enders et al. [58] described the efficient asymmetric total synthesis of (-)-callystatin A, a potent cytotoxic polyketide from a marine sponge, employing a combination of chemical and biocatalytic methods. Therefore, *tert*-butyl 6-chloro-3,5-dioxohexanoate was subjected to dried baker's yeast in a biphasic system (water/XAD-7 adsorber resin) and was regio- and enantioselectively reduced to the (5*R*)-hydroxy keto ester in 94% enantiomeric excess [58]. Furthermore, Bertau and Burli [59] reported the application of *Saccharomyces cerevisiae *cells for the synthesis of (2*S*,5*S*)-hexanediol. Glucose was used as auxiliary substrate for cofactor regeneration and with 25 mmol of hexanedione as substrate complete conversion with > 99% ee, 96% de and 75% yield was achieved. Servi and coworkers [60] showed that whole cells of *Geotrichum candidum *CBS 233.76 (95% yield, > 98% ee; 4 g/L, with absorbing resin Amberlite XAD-1180) and *Rhodotorula mucillaginosa *CBS 2378 (88% yield, > 99% ee; 1 g/L) reduced 3,4-dichlorophenacyl chloride to the (*S*)- and (*R*)-alcohol, respectively.

Matsuyama et al. [61] screened several yeast strains for the large scale production of (*R*)-1,3-butanediol, as the productivity of already reported asymmetric microbial reduction processes (for example with *S. cerevisiae*) was not satisfactory. Starting with 4-hydroxy-2-butanone, *Candida arborea *IAM 4147 and *Issatchenkia scutulata *IFO 10070 showed excellent ee (99%) but low yield, whereas *Kluyveromyces lactis *IFO 1267 gave high yield and good enantiomeric excess (93%) for (*R*)-1,3-butanediol. In contrast, *Candida parapsilosis *IFO 1396 performed the best for (*S*)-1,3-butanediol production (98% ee, 60% yield). In recent times, a new yeast isolate, namely *Candida tropicalis *PBR-2 MTCC 5158 was reported to enantioselectively reduce acetophenone and several substituted analogues to the corresponding (*S*)-alcohols with an enantiomeric excess of > 97%, mostly even > 99% [62]. As the natural aroma compounds 2-phenylethanol (2-PE) and 2-phenylethylacetate (2-PEAc) are of high industrial value, Etschmann and Schrader [63] lately improved their production by employing *Kluyveromyces marxianus *CBS 600 for a growth-associated product formation starting with L-phenylalanine. 26.5 g L^-1 ^2-PE and 6.1 g L^-1 ^2-PEAc were obtained in the organic phase which consisted of polypropylene glycol 1200, used as *in situ *extractant. This corresponded to space-time yields of 0.33 (2-PE) and 0.08 g L^-1 ^h^-1 ^(2-PEAc) surpassing the results of a previously reported *S. cerevisiae *process (Table 1; Additional File 1) [64]. Finally, Nakamura et al. [41] reviewed the use of *Geotrichum candidum *whole-cell preparations for the synthesis of chiral secondary alcohols with high enantioselectivities (up to > 99% ee).

In addition to these and also future findings in the field of selective yeast carbonyl-bioreductions, a strong trend for the construction of so-called 'designer-bugs' namely whole-cell biocatalysts co-expressing all required enzymes can be observed [65]. Most often *E. coli *is employed as recombinant host [66-69] due to the wealth of oxidoreductases found in yeasts, in particular, in *Saccharomyces cerevisiae*. Nowadays, the increasing number of annotated DNA sequences helps to identify enzymes involved in bioreductions and provides the information needed for the rational design of recombinant whole-cell biocatalysts [47].

#### 1.2 Reduction of C=C-bonds

**Figure 2:**
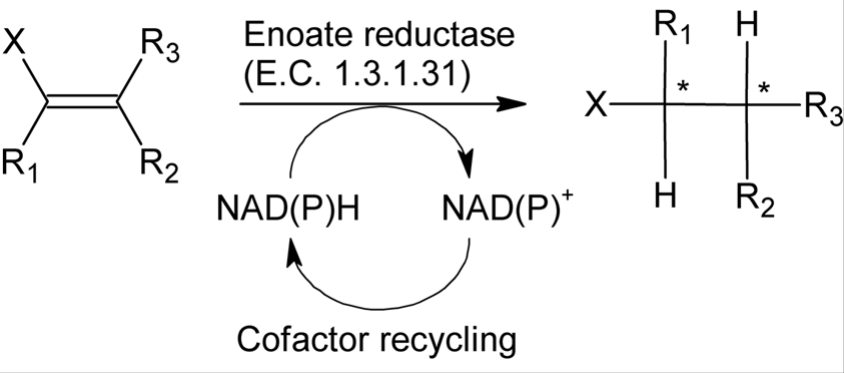
Enoate reductases perform the NAD(P)H-dependent, asymmetric bioreduction of activated C=C bonds. A cofactor recycling system is required for an economic process. Asterisks (*) indicate chiral centers, X depicts an activating group such as carbonyl-, carboxyl-, imide- or nitro-group [24,71].

In 1933, the first flavin-dependent redox enzyme was discovered in Brewer's bottom yeast by Warburg and Christian [70]. Enzymes of this family are also known as 'old yellow enzymes' because of their color derived from the flavin cofactor. Typical substrates [24,71] are alkenes which are 'activated' by electron-withdrawing substituents. Such substrates are reduced at the expense of NAD(P)H leading to enantiomerically pure alkanes. Thereby up to two chiral carbon centers are created (Figure 2).

In the past, most asymmetric bioreductions of activated C=C bonds employing enoate reductases were performed with whole cells, since problems with external cofactor recycling and the enzyme's sensitivity to traces of oxygen were encountered [24,72]. With whole-cell bioreductions often excellent stereoselectivities were achieved. However, chemoselectivity is often poor, regarding especially competitive C=C- and C=O-bond reductions. Whole-cells do not only provide enoate reductases but also alcohol dehydrogenases which both depend on the same nicotinamide cofactor. Uncoupling is hardly possible and the relative rates of alcohol dehydrogenases are comparable to those of enoate reductases leading to undesired by-products [71]. Nevertheless, successful examples for asymmetric bioreductions of α,β-unsaturated ketones exist, for example, with *S. cerevisiae *cells: Careful reaction control during oxoisophorone bioreduction led to the main product ((*R*)-2,2,6-trimethylcyclohexane-1,4-dione) in > 80% yield. The unwanted by-products were kept to a minimum. The product – also known as (*R*)-levodione – was produced on a 13 kg-scale [73]. It is industrially used for 3-hydroxycarotenoid production [74] (Table 2 and Additional File 2).

In general, the most prominent whole-cell biocatalyst employed was again baker's yeast (*S. cerevisiae*) which was tested with a great variety of differently substituted alkene substrates, such as e.g. different α,β-unsaturated nitroalkenes. It tolerated many different functional groups like alkyls and (substituted) aryls attached to the nitroalkene moiety [75,76]. However, also alternative yeasts were described to be active. *Geotrichum candidum *[77], *Rhodotorula rubra *[78] and *Rhodosporidium *sp. [78] were found to possess enoate reductases, active for diverse non-natural α,β-unsaturated carbonyl- and carboxyl-compounds, respectively [71]. *Candida *sp., *Rhodotorula *sp. and *Torulopsis *sp. were also shown to be active on α,β-unsaturated nitroalkenes [79]. Currently, the use of isolated enzymes or the construction of 'designer bugs' provides the possibility to reduce side reactions significantly. Desired yeast enoate reductases (e.g. from *Saccharomyces cerevisiae *[80], *Saccharomyces carlsbergensis *[81] or *Candida macedoniensis *[82]) were recently cloned and overexpressed in *E. coli*. Kataoka et al. [82] even coexpressed glucose dehydrogenase (GDH) for efficient cofactor regeneration. More details were outlined and reviewed by Faber [24] and Stuermer et al. [71], respectively.

#### 1.3 Oxidation and racemization reactions

Yeast alcohol oxidases were shown to be responsible for the oxidation of methanol and other primary alcohols [83-85]. However, they did not oxidize secondary alcohols. In general, reports on oxidation reactions performed by yeasts are quite rare. Considering especially the oxidation of secondary alcohols, one has to recognize that instead of creating a chiral center, it is 'destructed'. The reaction is therefore regarded to be of limited synthetic use except for the regioselective oxidation of polyols where chemical methods are often inadequate [24]. However, the recent, increasing demand for 'deracemization processes', leading to a single stereoisomer in 100% yield, evoked the need for 'clean' racemization protocols [86]. Not to forget, the oxidation of sulfides can result in chiral sulfoxides which have been widely used in organic synthesis as asymmetric auxiliary groups to control the stereochemical outcome of the reaction at nearby centers [87,88].

In 1979, Patel et al. [89] described the application of cell suspensions of *Candida utilis *ATCC 26387, *Hansenula polymorpha *ATCC 26012, *Pichia *sp. NRRL-Y-11328, *Torulopsis *sp. and *Kloeckera *sp. for the oxidation of secondary alcohols. 2-Propanol, 2-butanol, 2-pentanol, and 2-hexanol were oxidized to the corresponding methyl ketones. Furthermore, *Saccharomyces cerevisiae *was reported to catalyze the selective oxidation of sulfides to sulfoxides. Beecher et al. [90] investigated the oxidation of *p*-tolyl sulfide to the *R*-sulfoxide (92% ee) which was used for the preparation of the mevinic acid-type hypocholestemic agent. Buist et al. [91] employed baker's yeast for the enantioselective sulfoxidation of a fatty acid analogue (~70% ee). In this study, the authors presumed that a desaturase could be responsible for the transformation, but did not verify it.

In order to satisfy needs for sophisticated racemization protocols, Nestl et al. [86,92] screened various microbial cells for the biocatalytic racemization of functionalized α-hydroxyketones. Although whole lyophilized cells of *Geotrichum candidum *DSM 6401, *Candida parapsilosis *DSM 70125, or *Kluyveromyces lactis *DSM 3795 were identified to racemize a variety of the employed target substrates, the activities of yeast strains were modest compared with bacteria and fungi. Traces of diketones as 'side products' let them assume that dehydrogenase enzymes were responsible for the racemization reaction [86].

Matsuyama et al. [61] performed the oxidative kinetic resolution for the large-scale production of (*R*)-1,3-butanediol employing *Candida parapsilosis *IFO 1396. Starting with 20 kg (*rac*)-1,3-butanediol, 258 kg cells, 465 kg water and 7.5 kg calcium carbonate resulted in ~3.1 kg of (*R*)-1,3 butanediol in high chemical (~99%) and optical purity (94% ee). A subsequent enzyme purification step elucidated (*S*)-1,3-butanediol dehydrogenase (*Cp*SADH) to be responsible for producing (*R*)-1,3-butanediol from the racemate. *Cp*SADH was then also overexpressed in *E. coli *yielding in increased specific activities and a strain with good racemization properties for the production of (*R*)-1,3-butanediol (95% yield, 94% ee) without the need for cofactor regeneration. Recently, Titu and Chadha [93] described the biocatalytic preparation of optically pure alkyl 3-(hetero-2-yl)-3-hydroxypropanoates by deracemization. *Candida parapsilosis *ATCC 7330 was employed and various optically pure products resulted in high ee (89–99%) and yields (58–75%). These compounds represent important chiral intermediates for the synthesis of pharmaceuticals such as duloxetine, tetrahydropyrans or heteroarylaminoalkanols, among others.

In most cases, however, the enzyme or enzyme-set catalyzing racemization reactions remains unknown. Their identification and employment in a non-natural environment (biotransformation with solubilized or immobilized enzyme(s) or a recombinant whole-cell biocatalyst) could further reduce the amount of undesired side-reactions, improve optical purities and also provide a platform for enzyme engineering approaches. Examples of 'yeast designer bugs' with enzymes for chiral sulfoxide production are given in Section 3.

#### 1.4 Hydrolase reactions

*Saccharomyces cerevisiae *was also tested for hydrolase reactions. However, first yeast-catalyzed deacylation reactions were regarded to be undesired side-reactions when Mamoli et al. [94] was studying whole cells of baker's yeast for the stereoselective reduction of estrogen ester. Later, the whole variety of hydrolysis reactions performed by *Saccharomyces cerevisiae *was investigated and comprehensive reviews on the properties of proteinases [95], lipases and esterases [34] were given. What was encountered quite fast was the complex reaction control when whole microbial cells were employed [24]. To overcome the problems resulting from the metabolism of fermenting *Saccharomyces cerevisiae*, lyophilized resting cells were employed. Thus, for example the acetate of pantolactone – the chiral precursor for vitamin B_5 _synthesis – was nicely resolved [96]. In addition, differently substituted 1-alkyn-3-yl acetates were converted with high ee (91–96%) to the corresponding (*S*)-alcohols and acceptable enantioselectivities (E ~46–100) were reached [24].

Many yeast hydrolases especially from *Candida *sp. were employed in industrial biotransformations. However, they were mostly used as immobilized enzyme preparations due to perturbing unspecific hydrolases available in whole cells. Only were other fungal and some bacterial strains industrially employed as whole cells for hydrolase reactions [74]. Immobilized whole cells of *Fusarium oxysporum *were for example used for the production of D-pantoic acid starting with (*rac*)-pantolactone [97]. Suspended whole cells of *Comamonas acidovorans *catalyzed the conversion of (*rac*)-2,2-dimethylcyclopropanecarboxamide to the corresponding (*R*)-carboxylic acid and the (*S*)-carboxamide remained [98]. In addition, whole cells of *Bacillus brevis *[99,100], *Pseudomonas *sp. [74] and *Arthrobacter *sp. [101] were employed i.a. for hydantoinase catalyzed reactions.

Furthermore, *Rhodotorula *sp., *Rhodosporidium *sp. and *Trichosporon *sp. were found to give best enantioselectivities for the hydrolysis of monosubstituted oxiranes. All showed preferred enantiopreference for the (*R*)-form and regioselectivity for the sterically less hindered carbon atom [24]. Recently, again a highly selective epoxide hydrolase from *Rhodosporidium paludigenum *CBS 6565 was observed during whole-cell biotransformations, subsequently cloned and further characterized [102].

#### 1.5 Formation of C-C-bonds

From a synthetic point of view, carbon-carbon bond forming reactions are highly interesting as new asymmetric carbon centers can be formed. However, this type of yeast whole-cell biotransformation was and is limited to only a few examples [30,34]. The most prominent and industrially employed acyloin condensation performed by *Saccharomyces cerevisiae *yields in the formation of (1*R*)-phenylacetyl carbinol, a chiral synthon of D-ephedrine (Table 2 and Additional File 2). Crout et al. [103] succeeded in finding out the detailed reaction pathway, involving pyruvate decarboxylase [104]. Not only benzaldehyde can be subjected to baker's yeast mediated acyloin condensations. Fuganti et al. [105,106] also investigated the conversion of α,β-unsaturated aldehydes giving optically active diols and used this type of reaction for the production of the C-14 chromanyl moiety of α-tocopherol (vitamin E) [107]. An interesting L-threonine aldolase was isolated from *Candida humicola*. However, only the isolated enzyme was characterized [108].

### 2. Application of yeast whole-cell biocatalysis in industry

One of the first industrial processes combining microbiological and chemical synthesis was the acyloin-type condensation [109] of benzaldehyde resulting in (1R)-phenylacetylcarbinol which is subsequently converted to (1R, 2S)-ephedrine and (1R, 2S)-pseudoephedrine, respectively. For that purpose, *Saccharomyces cerevisiae *whole cells were used in an aqueous medium [110]. The products find application for the treatment of asthma, hay fever and as a bronchodilating agent and decongestant [74,110].

In addition to this application, two more lyase-based biocatalytic approaches with whole cells were employed in industry: *Candida rugosa *enoyl-CoA hydratase catalyzes for example one of three biotransformation steps from butyric acid to (*R*)-β-hydroxy-n-butyric acid [100,111], namely the enantioselective introduction of a hydroxy-group at the β-position of the α,β-unsaturated acid which resulted after dehydrogenation. The chiral product is produced with high optical purity (> 98% ee) and a space-time yield of 5–10 g·L^-1^·d^-1^. It is used for the production of a carbapenem intermediate [74]. The same whole-cell biocatalyst is also capable of converting isobutyric acid to (*R*)-β-hydroxy-isobutyric acid, in this case with 98% yield, 97% ee and again a space-time yield of 5–10 g·L^-1^·d^-1 ^[100,111,112]. (*R*)-β-hydroxy-isobutyric acid is a chiral synthon for the synthesis of captopril, an inhibitor of angiotensin converting enzyme [74]. Secondly, suspended whole cells of *Rhodotorula rubra *possess an L-phenylalanine ammonia lyase which performs the selective transformation of *trans*-cinnamic acid and ammonia to L-phenylalanine. The reaction is performed in aqueous solution at 25°C and pH 10.6 and results in 85.7% yield. The L-phenylalanine is commonly applied for the production of artificial sweeteners like aspartame and in parenteral nutrition. It can also be used for the synthesis of macrolide antibiotics such as rutamycin B [74,99,100].

Most yeasts which are industrially employed for whole-cell biocatalysis however are used for asymmetric reductions. Eli Lilly for example employed whole cells of *Zygosaccharomyces rouxii *for the enantioselective reduction of 3,4-methylenedioxy-phenylacetone to the corresponding (*S*)-alcohol. Since the substrate was toxic to the microorganism, it was provided in an adsorbed form on the resin XAD-7. This allowed elevated reaction concentrations and high levels of volumetric productivity. The process showed a space-time yield of 75 g·L^-1^·d^-1 ^and a high optical purity (> 99.9% ee) [113-116]. The strain *Geotrichum candidum *SC5469 was described to be employed by Bristol-Myers Squibb for the stereoselective reduction of 4-chloro-3-oxo-butanoic acid methyl ester to the corresponding (*S*)-3-hydroxy-alcohol [74]. This conversion yielded the product in 95% yield and 99% ee and thus provided a useful chiral building block, e.g. for the synthesis of a cholesterol antagonist that inhibits hydroxymethyl glutaryl CoA reductase [117,118]. Furthermore, a second *Candida *sp., namely *Candida sorbophila*, was employed for the production of a chiral (*R*)-amino alcohol (Entry 3 in Table 2 and Additional File 2) with high optical purity (> 98% ee; 99.8% after purification) and high chemical purity (95%) [119]. The resulting (*R*)-amino alcohol is an important chiral synthon for the synthesis of a β-3-agonist which can be used for obesity therapy and the treatment of associated type II diabetes, coronary artery disease and hypertension [74,120].

**Table 2:** Industrial biotransformations [74] employing wild type yeast whole-cell biocatalysts ordered by E.C. numbers

**Yeast strain**	**Enzyme name (E.C. No.)**	**Reaction**	**Product/*Application***	**Company**
*Zygosaccharomyces rouxii*	Alcohol NAD^+ ^oxidoreductase (1.1.1.1)		LY 300164 = Benzodiazepine/***tested for treating amyotrophic lateral sclerosis***	Eli Lilly and Company, USA

*Geotrichum candidum*	Dehydrogenase, NADPH-dependent (1.1.X.X)		chiral β-hydroxy ester/***precursor for cholesterol antagonist inhibiting HMG-CoA reductase***	Bristol-Myers Squibb, USA

*Candida sorbophila*	Dehydrogenase (1.1.X.X)		(*R*)-amino alcohol/***intermediate for a β-3-agonist used for obesity therapy, and to decrease hypertension, coronary artery disease and the level of associated type II diabetes***	Merck & Co, Inc., USA

*Pichia methanolica*	Reductase (1.1.X.X)		Ethyl-5-(*S*)-hydroxyhexanoate and 5-(*S*)-hydroxyhexanenitrile/***chiral building blocks***	Bristol-Myers Squibb, USA

*Candida boidinii *(**E2**) [and *E. coli *with cloned **E1**]^a ^or *Pichia pastoris *(**E2 **+ cloned **E1**)^b^	**E2**: FDH (1.2.1.2) [**E1**: PheDH (1.4.1.20) from *Thermoactinomyces intermedius*]		(*S*)-2-Amino-5-(1,3-dioxolan-2-yl)-pentanoic acid/***one building block for Omapatrilat synthesis (ACE and NEP inhibitor)***^c^	Bristol-Myers Squibb, USA

*Trigonopsis variabilis *ATCC 10679	D-Amino acid oxidase (1.4.3.3)		L-6-Hydroxynorleucine/***chiral intermediate for synthesis of vasopeptidase inhibitor and other antihypertensive metalloproteinase inhibitors***	Bristol-Myers Squibb, USA

Baker's yeast (= *Saccharomyces cerevisiae*)	Reductase (1.X.X.X)		***The corresponding dione is an intermediate for the synthesis of natural 3-hydroxycarotenoids (e.g. cryptoxanthin, zeaxanthin) and other terpenoid compounds*.**	Hoffmann La-Roche, CH

*Cryptococcus laurentii *(**E1**) [and *Achromobacter obae *(**E2**)]^a^	**E1**: Lactamase (3.5.2.11) and [**E2**: Racemase (5.1.1.15)]^a^		L-Lysine/***nutrient and food supplement***	Toray Industries Inc., Japan

*Saccharomyces cerevisiae*	Pyruvate decarboxylase (4.1.1.1)		PAC → ephedrine and pseudoephedrine/***treatment of asthma, hay fever, and used for bronchodilating agent and decongestant***	Krebs Biochemicals & Industries Ltd., India

*Candida rugosa*	Enoyl-CoA hydratase (4.2.1.17)		(*R*)-β-Hydroxy-n-butyric acid and (*R*)-β-Hydroxy-isobutyric acid/***chiral synthons for carbapenem intermediate and captopril (ACE-inhibitor)^c^, respectively***	Kanegafuchi Chemical Industries Co., Ltd., Japan

*Rhodotorula rubra*	L-Phenylalanine ammonia-lyase (4.3.1.5)		L-phenylalanine/***artificial sweetener aspartame, parenteral nutrition, chiral building block for Rutamycin B synthesis***	Genex Corporation, USA

^a^Bacterial whole-cell biocatalyst. ^b^In this case, also engineered *P. pastoris *cells were employed. ^c^ACE = angiotensin-converting enzyme; NEP = neutral endopeptidase.

The yeast *Pichia methanolica *was applied by Bristol-Myers Squibb because of its beneficial reductase for the reduction of ethyl 5-oxo-hexanoate and 5-oxo-hexanenitrile to the corresponding (*S*)-alcohols. The reaction resulted in high yield (90–95%) and high enantiomeric excess (> 95%) [121].

Chiral intermediates for the production of the antihypertensive drug, Omapatrilat, were produced by two different biocatalytic approaches (Entry 5 in Table 2 and Additional File 2 ): In both cases the enzyme phenylalanine dehydrogenase from *Thermoactinomyces intermedius *(*Ti*PheDH) was employed for the reductive amination step and a yeast formate dehydrogenase (FDH) was required for NADH regeneration. For the first, heat-dried cells of recombinant *E. coli *with cloned *Ti*PheDH were used together with heat-dried *Candida boidinii *cells, as a source of FDH. The conversion resulted in 96% yield and > 99% ee. Secondly, dried recombinant *Pichia pastoris *cells containing endogenous FDH and overexpressing *Ti*PheDH were employed. The application of the second biocatalyst resulted in slightly improved yield (97.5%) and also high enantiomeric excess (> 99%) for the production of (*S*)-2-amino-5-(1,3-dioxolan-2-yl)-pentanoic acid [122]. Regarding cofactor regeneration, NAD^+ ^produced during the reaction was in both cases regenerated to NADH by the oxidation of the auxiliary substrate formate to CO_2 _[123].

A resolution technique is the basis for the whole-cell biocatalytic application of *Trigonopsis variabilis *ATCC 10679 cells. (*rac*)-6-Hydroxynorleucine, which results after hydrolysis of commercially available 5-(4-hydroxybutyl)-hydantoin, is converted to the ketoacid leaving the L-enantiomer. This is then isolated by ion exchange chromatography and can be used as chiral synthon, again, for the synthesis of Omapatrilat [43], the production of a vasopeptidase inhibitor, and the synthesis of C-7 substituted azepinones which represent potential intermediates for other antihypertensive metalloproteinase inhibitors [74]. The remaining 2-keto-6-hydroxyhexanoic acid was further converted to L-6-hydroxynorleucine by reductive amination catalyzed by beef liver glutamate dehydrogenase (89–92% yield, > 99% ee, 3 h) [124]. *Bacillus megaterium *glucose dehydrogenase was used for NAD^+ ^recycling.

Furthermore, *Saccharomyces cerevisiae *was industrially employed because of its ene-reductase which belongs to the old yellow enzyme family. The substrate oxoisophorone was subjected to fermentative reduction with baker's yeast in an aqueous solution of saccharose. Periodically, substrate and saccharose were added in order to avoid toxic levels of oxoisophorone. The product 2,2,6-trimethylcyclohexane-1,4-dione resulted in 80% yield and < 97% ee and was used as intermediate for the synthesis of natural 3-hydroxycarotenoids such as zeaxanthin, cryptoxanthin and other structurally related compounds. The space-time yield of the process was given with 2.8 g·L^-1^·d^-1 ^[73,74].

Finally, a hydrolase-based reaction employing whole cells of *Cryptococcus laurentii *was used by the Japanese company Toray Industries Inc. for the synthesis of L-lysine [99,100,125]. For that purpose actually a combination of cells from *Cryptococcus laurentii *with intrinsic L-lysine lactamase and the bacterium *Achromobacter obae *with a lactame racemase were used. L-Lysine resulted with > 99.5% ee and was applied as nutrient and food supplement. Alternatively, the combination of cells from the yeast *Candida humicola *and the bacterium *Alcaligenes faecalis *were used. However, this process was totally replaced by highly efficient fermentation methods [74]. All above described industrial applications of yeast whole-cell biocatalysts are depicted in Table 2 and Additional File 2.

### 3. Recombinant Yeasts for Whole-Cell Biocatalysis

Remarkable progress in the field of genetic engineering made a variety of yeast strains, in particular *Saccharomyces cerevisiae*, susceptible to modifications by recombinant DNA technology. In the mid-1980s, a number of expression systems based on alternative yeasts appeared which eliminated some initial limitations to *S. cerevisiae *such as low yields or a high extent of undesirable hyperglycosylation. Advantages and disadvantages of such 'nonconventional' yeasts for recombinant protein production were reviewed in detail elsewhere [126-130]. Prominent established examples are *Hansenula polymorpha *[131], *Pichia pastoris *[132-134], *Kluyveromyces lactis *[135], *Yarrowia lipolytica *[136], *Candida boidinii *[128], and *Schizosaccharomyces pombe *[137], among others.

In order to circumvent disadvantages of wild-type whole-cell biocatalysts, different groups started to construct 'designer yeasts' to improve first of all single-step biocatalytic syntheses. Methods for gene knockout and overexpression of heterologous enzymes, offered alternative approaches to classical techniques aiming at improving the whole-cell biocatalyst's selectivity (see Section 2). Catalytic activities of competing host-own enzymes were eliminated and desired catalytic properties could be reinforced, respectively. First, simple 'single mutants' – with one deleted or overexpressed gene – were thoroughly studied but soon combinatorial approaches enlarged the spectrum of valuable whole-cell biocatalysts. Of course, the identification of complete genome sequences facilitated investigations and elucidated the wealth of the host-own enzyme spectrum. Since the publication of the complete genome sequence of *S. cerevisiae *in 1996 [48,49], more than 30 yeast genomes have been completely or partly sequenced and deposited in public databases (e.g. NCBI: ). This information is now available and ready to be exploited for the construction of valuable whole-cell factories for i.a. biocatalytic applications.

At the same time, a second branch of science, namely 'metabolic engineering', emerged in the field of genetic engineering and in 1991, Bailey [138] extensively reviewed this new field for the first time. In the following years, metabolic engineering has developed rapidly and consequently resulted in various reviews [139-142] and textbooks [143,144] explaining basic principles and methodologies. The many existing examples of metabolic engineering were classified in several categories which include heterologous protein production, extension of substrate range, pathways leading to new products, pathways for the degeneration of xenobiotics, engineering of cellular physiology for process improvement, elimination/reduction of by-product formation and improvement of yield or productivity [142]. All approaches have two important parts in common: first, the identification of the most promising target(s) for genetic manipulation by solid strain analysis and second, the construction of genetically modified cells [141]. As several categories of metabolic engineering and the construction of recombinant strains for whole-cell biocatalysis target at improving similar characteristics of a cell, it is not always possible to draw a clear line between these two disciplines. However, the variety of recent reports on metabolic engineering of yeasts for the efficient production of top value added chemicals (e.g. ethanol, glycerol, xylitol, succinic acid and other organic acids) [145] would go beyond the scope of this review. Therefore, we refer to existing comprehensive overviews in this field of research [141,142,146-150].

In the following we focus on engineered yeast platform strains for the production of mostly chiral precursors for the pharmaceutical, food or feed industry, thereby including single- and multi-step biocatalytic reactions. Finally, we highlight recent pathway engineering efforts leading to structurally complex natural products, a field which indicates the possible catalytic scope of engineered yeasts.

#### 3.1 Organic single or few-step transformations catalyzed by engineered yeast cells

Employing non-engineered yeast strains for whole-cell biotransformations has often resulted in low chemical and optical purities. As selective reduction and oxidation reactions are the most thoroughly investigated applications of whole-cell yeast biocatalysts, the first recombinant yeast strains were also engineered for this class of biotransformations. Several groups subjected *S. cerevisiae *to step-by-step gene knockout and overexpression of various reductases and studied the catalytic properties of the resulting engineered yeast strains in detail (Table 3 and Additional File 3).

**Table 3:** Examples for one- to two step enzymatic reactions employing engineered yeast whole-cell biocatalysts (+ overexpression; - knockout/deletion).

**Whole-Cell Biocatalyst**	**Engineering** **+ overexpression** **- deletion**	**Substrate**		**Product**	**Performance: yield (ee)**		**Ref.**
*Saccharomyces cerevisiae*	- β-keto reductase				53% (90%) (slow, continuous substrate feed)		[151]

*Saccharomyces cerevisiae*	A: +Fasp, -Gre2p, -Ypr1p^IV^				A:	d: 78% (91% *R*)^II^	[152]
	B: -Fasp, +Gre2p^IV^					e: 26% (> 98% *RI*)^I^	
			a: R_1 _= Me; R_3 _= Me		B:	a: 76% (> 98% *S*)^I^	
			b: R_1 _= Et; R_3 _= Me			b: 85% (> 98% *S*)^I^	
			c: R_1 _= Me; R_3 _= Et			c: 83% (> 98% *S*)^I^	
			d: R_1 _= Et; R_3 _= Et			d: 87% (> 98% *S*)^I^	
			e: R_1 _= *n*-Pr; R_3 _= Et			e: 90% (> 98% *S*)^I^	

*Saccharomyces cerevisiae*	A: - Gre2p, + Ypr1p^IV^		a: R_2 _= Me		A:	a: 90% (98% *syn*)^II^	[152]
	B: + Gre2P, - Ypr1p^IV^		b: R_2 _= Et			b: 89% (83% *syn*)^II^	
			c: R_2 _= allyl			c: 92% (65% *syn*)^II^	
			d: R_2 _= propargyl			d: 75% (> 98% *syn*)^II^	
					B:	a: 86% (70% *syn*)^I^	
						b: 73% (67% *anti*)^I^	
						c: 67% (> 98% *anti*)^I^	
						d: 70% (> 98% *anti*)^I^	

*Saccharomyces cerevisiae*	A: +Ymr226c^III^					A: 72% (87%, *S*)^I^	[153,154]
	B: +Ara1p^III^		R_1 _= Me; R_2 _= Me			B: 48% (91%, *S*)^I^	
	C: +Ypr1p^III^					C: 62% (87%, *S*)^I^	

*Saccharomyces cerevisiae*	+ reductase gene YDR368w^V^	bicyclo[2.2.2]octane-2,6-dione		(1*R*,4*S*,6*S*)-6-hydroxy-bicyclo[2.2.2]octane-2-ol		97% de	[155]
						> 99% ee	
						84% yield	

*Saccharomyces cerevisiae*	+ multiple copies of *Candida tenuis *xylose reductase variant W23F		a: R = H			a: 35% (> 99.9%, *S*)^VI^	[158]
			b: R = Ph			b: 80% (> 99.9%, *R*)^VI^	
			c: R = *o*-Cl-Ph			c: 76% (99.6%, *R*)^VI^	
			d: R = *m*-Cl-Ph			d: 14% (> 99.9%, *R*)^VI^	
			e: R = *p*-Cl-Ph			e: 74% (99.9%, *R*)^VI^	
			f: R = *p*-CN-Ph			f: 100% (99.4%, n.d.)^VI^	

*Saccharomyces cerevisiae*	+ *Antirrhinum majus *benzoic acid methyltransferase (BAMT)					~1 mg methyl benzoate per L of culture (with OD_600 _= 1), after 24 h	[160]

*Pichia pastoris*	+ *Rhodotorula glutinis *epoxide hydrolase					~36% (> 98%) (theoretical yield: 50%)	[171]

*Schizosaccharomyces pombe*	+ human CYP11B1-V78I + electron transfer proteins adrenodoxin (Adx) & adrenodoxin reductase (AdR)					2.5-fold higher activity compared with parental strain	[172]

*Schizosaccharomyces pombe*	+ human CYP2D6					56% yield 98.5% purity	[173]

*Schizosaccharomyces pombe*	+ *Trigonopsis variabilis *D-Amino Acid Oxidase (multi copies) - catalase	Cephalosporin C		α-ketoadipyl-7-cephalosporanic acid and glutaryl-7-amino-cephalosporanic acid		550 U/g CDW increased mechanical resistance	[191]

^I^carbon source: glucose

^II^carbon source: galactose

^III^Ara1p = *S. cerevisiae *reductase, NADPH-dependent; Ypr1p = *S. cerevisiae *reductase, NADPH-dependent; YMR226c = *S. cerevisiae *short chain dehydrogenase ORF [153].

^IV^Fasp = *S. cerevisiae *fatty acid synthase; Gre2p = *S. cerevisiae *α-acetoxy ketone reductase; Ypr1p = *S. cerevisiae *aldo-keto reductase [152].

^V^*S. cerevisiae *2-methylbutyraldehyde reductase (NCBI accession number: NP 010656)

^VI^Whole-cell bioreductions of α-keto esters (10 mM) under anaerobic conditions using 1 M ethanol as co-substrate; n.d. = not determined.

In 1985, Shieh et al. [151] already succeeded in improving the optical purity for ethyl (*R*)-4-chloro-3-hydroxybutanoate production starting with the prochiral β-keto ester. They employed the *S. cerevisiae *ATCC 26403 mutant lacking a competing β-keto reductase and achieved 90% ee compared to 57% ee for the wild-type strain [151]. Fifteen years later, Rodríguez et al. [152] altered the levels of three *S. cerevisiae *oxidoreductases, namely fatty acid synthase (Fasp), aldo-keto reductase (Ypr1p), and α-acetoxy ketone reductase (Gre2p), respectively. They first created a set of 'first-generation' yeast strains deleting or overexpressing only one single enzyme at a time. Based on subsequent results, a 'second-generation' set of yeast strains was constructed, combining gene deletion and overexpression. Several β-keto esters were used as substrates and among the engineered strains not only significantly improved stereoselectivities compared to the wild-type but also engineered strains with inverted selectivities were found [152] (see for example Table 3 and Additional File 3, Entry 2, Substrate d and e). However, this study also revealed that additional yeast proteins exist which might participate in the reduction of some of the subjected β-keto ester substrates and deteriorate especially enantiopurities. Later, forty-nine open reading frames encoding for potential reductase activities were elucidated in the complete *S. cerevisiae *genome [68]. However, up to now not all corresponding reductases have been investigated in detail. Some selective alternative *S. cerevisiae *reductases were identified and examined by Gorwa-Grauslund and coworkers [153,154]. Overexpressing the NADPH-dependent reductases Ara1p, Ypr1p and YMR226c, respectively, led to yeast strains with increased yield (≥ 48%) and enantiomeric excess (≥ 87%) for the conversion of diacetyl to (*S*)-acetoin (wild-type: 42% yield, 80% ee) [153,154]. Furthermore, Johanson et al. [155] improved the asymmetric reduction of a bicyclic diketone to (1*R*,4*S*,6*S*)-6-hydroxy-bicyclo[2.2.2]octane-2-one which is used as intermediate for the production of transition metal-based chiral chemical catalysts [156]. A combination of reaction and strain engineering (overexpression of the *S. cerevisiae *reductase YDR368w in *S. cerevisiae *– Table 3 and Additional File 3) led to 84% yield, > 99% ee and 97% de compared to initially described results with 52% yield, 95% ee, and 84% de – after chromatography and crystallization [157]. Recently, Kratzer et al. [158] succeeded in overcoming limitations of baker's yeast in the asymmetric reduction of α-keto esters using also a combinatorial approach. They improved the enzyme by exchanging the amino acid residue tryptophane 23 with phenylalanine (CtXR-W23F) which resulted in an up to eightfold higher NADH-dependent activity compared to the wild-type enzyme employing a series of aromatic α-keto esters as substrates [159]. The strain *S. cerevisiae *was engineered to efficiently overexpress CtXR-W23F by introducing multiple copies of the expression cassette (strain *S.c. *WF2μ). Finally, the reaction itself was optimized performing it under anaerobic conditions in the presence of EtOH. Thereby NADH-regeneration was preferred. This was accomplished by the yeast's own NADH-dependent alcohol dehydrogenase (ADH) and NAD(P)H-dependent aldehyde dehydrogenase (AlDH). Thus, the action of endogenous NADPH-dependent yeast reductases with diverse enantiopreferences was suppressed [158]. α-Hydroxy esters were obtained in acceptable yields and high enantiopurities (≥ 99.4% ee, see Table 3 and Additional File 3, Entry 6).

Exploiting the advantage of *S. cerevisiae *to be 'Generally Regarded As Safe' (GRAS) for human consumption, Farhi et al. [160] engineered this yeast for the improved synthesis of the food flavoring methyl benzoate. This was achieved by expressing the *Antirrhinum majus *benzoic acid methyltransferase (BAMT) under the control of the copper-inducible *CUP1 *promoter. A yield of ~1 mg methyl benzoate per liter of culture was achieved after 24 h, starting with benzoic acid. In addition, it was demonstrated that yeast strains expressing BAMT were more tolerant towards benzoic acid. Several groups also focused on engineering *S. cerevisiae *in order to modulate and improve the formation of antioxidants and aroma compounds during wine fermentation. In 2000, González-Candelas et al. [161] used a transgenic yeast strain for the improved production of resveratrol, an antioxidant which was reported to possess cancer chemoprotective properties [162,163]. They expressed the *Candida molischiana bglN *gene encoding for a β-glucosidase in the industrial wine yeast *S. cerevisiae *T73 (CECT1894) and yielded elevated contents of both *trans*- and *cis*-resveratrol (≥ 0.75 μM) compared to the wild-type (≥ 0.25 μM) [161]. Further engineering concepts for improved resveratrol production with recombinant yeast strains are discussed in detail in chapter 3.4. Subsequently, Genovés et al. [164] optimized the production of recombinant *Candida molischiana *BGLN enzyme in *S. cerevisiae *and tested the purified BGLN enzyme for vinification experiments. The efficient release of terpenes and alcohols from Muscat wine glycosides was observed. Smit et al. [165] tried to develop wine yeasts with optimized decarboxylating activity on phenolic acids. Therefore, two different phenolic acid decarboxylases (PADC from *Bacillus subtilis *and *p*-coumaric acid decarboxylase (PDC) from *Lactobacillus plantarum*) were expressed in *S. cerevisiae *under the control of the constitutive *S. cerevisiae *phosphoglyceratekinase I gene promoter and terminator (PGK1_P _and PGK1_T_). In most of the Chardonnay and Riesling wines, the vinification done by the recombinant strains resulted in slightly increased 4-ethylphenol, 4-vinylphenol and 4-vinylguaiacol concentrations. However, first results of industrial yeast fermentation analysis showed very high concentrations of volatile phenols close to and even higher than the ideal concentration. Thus, further improvements are necessary including a better regulation of the recombinant enzyme's overproduction or the pre-evaluation of the available concentration of precursor – namely phenolic acid components – in the grape juice. Cordente et al. [166] engineered *S. cerevisiae *in order to optimize concentrations of volatile esters which represent the largest and most important group of wine flavoring components produced during fermentation. They overexpressed the major mitochondrial and peroxisomal carnitine acetyltransferase (CAT2p) from *S. cerevisiae *but instead of increased levels of ester compounds reduced concentrations were observed. The authors hypothesized that the overexpression of Cat2p favors the production of acetylcarnitine and CoA and therefore limits the precursor for ester formation [166].

Finally, Stewart and coworkers tested a *Saccharomyces cerevisiae *strain expressing the cyclohexanone monooxygenase from the bacterium *Acinetobacter *sp. NCIB 9871 with a variety of substituted cycloalkanones [167-170] and several sulfides, dithianes and dithiolanes [88], respectively (Table 4 and Additional File 4). Thereby, they combined the broad substrate tolerance of the enzyme with baker's yeast tolerance for relatively high concentrations of organic compounds, providing a stable environment for the enzyme. Furthermore, no enzyme purification or additional NADPH-regeneration system was necessary and the enzyme from this potentially pathogenic bacterial strain (class II) [88] became easily accessible. Enzyme induction in the *Acinetobacter *strain also required the addition of cyclohexanol which complicated product isolation [68]. Table 4 and Additional File 4 depicts detailed results of enantioselective Baeyer-Villiger oxidations with the above described 'designer yeast'. Although high optical purities were achieved with the majority of investigated substrates, it is still clear that yields must be improved significantly in order to allow the biocatalyst's commercial application [68].

**Table 4:** Examples for one step enzymatic reactions employing the designer yeast made by Stewart and coworkers [88,167-170], namely *Saccharomyces cerevisiae *overexpressing the *Acinetobacter *sp. NCIB 9871 cyclohexanone monooxygenase. The data is chronologically ordered.

**Entry**	**Substrate**	**Product**	**Substrate/Product Code**	**Performance: yield (ee)**				**Ref.**
1			a: R = Me	a: 83% (≥ 98%)				[167,168]
			b: R = Et	b: 74% (≥ 98%)				
			c: R = Pr^i^	c: 60% (≥ 98%)				
			d: R = Pr	d: 63% (92%)				
			e: R = allyl	e: 62% (95%)				

					1		2	
					
2			a: R = Me	a:	50% (49%)		---^I^	[168,169]
			b: R = Et	b:	79% (95%)		69% (≥ 98%)	
			c: R = Pr^i^	c:	41% (≥ 98%)		46% (96%)	
			d: R = Pr	d:	54% (97%)		66% (92%)	
			e: R = allyl	e:	59% (≥ 98%)		58% (≥ 98%)	
			f. R = n-butyl	f:	59% (≥ 98%)		64% (98%)	

					1	2	3	
					
3			a: R = Me	a:	71% (≥ 98%)	60 % (≥ 98 %)	---^I^	[168]
			b: R = Et	b:	18% (70%)	20% (70%)	---^I^	
			c: R = Pr^i^	c:	N.R.^II^	---^I^	N.R.^II^	
			d: R = Pr	d:	11% (≥ 98%)	---^I^	8% (83%)	
			e: R = allyl	e:	15% (97%)	---^I^	9.3% (≥ 98%)	
			f. R = n-butyl	f:	37% (56%)	---^I^	11% (84%)	

					1		2	
					
4			a: R = n-Bu	a:	18% (≥ 98%)		32% (≥ 98%)	[170]
			b: R = n-Hex	b:	32% (≥ 98%)		42% (≥ 98%)	
			c: R = n-Oct	c:	25% (≥ 98%)		14% (≥ 98%)	
			d: R = n-	d:	39% (≥ 98%)		37% (≥ 98%)	
			C_11_H_23_					

					1^III^	2^III^	3^III^	
					
5			a: R = n-Pr	a:	27% (13%)	-^IV ^(33%)	-^IV ^(60%)	[170]
			b: R = n-Hex		overall yield: 44%; ratio 2 : 3 = 83 : 17			
					
				b:	54% (29%)	(60%)	-^IV^	
					overall yield: 20%; ratio 2 : 3 = > 99 : < 1			

6			a: R = Ph	a: 95% (> 99%)				[88]
			b: R = Bu^t^	b: 47% (99%)				
			c: R = Bu^n^	c: 53% (74%)				

7			a: R_1 _= H R_2 _= H	1a: 18% (90%) (2a: 19% yield)				[88]
			b: R_1 _= H R_2 _= Ph	1b: 30% (30%) (no sulfone 2b detected)				

8			a: R_1 _= H R_2 _= H	1a: 20% (75%) [2a: 45% yield]				[88]
			b: R_1 _= H R_2 _= CH_3_	1b: 16% (20%) [2b: 15% yield 76% ee]				
			c: R_1 _= H R_2 _= Ph	1c: 74% (20%) [no sulfone 2c detected]				

9			R_1 _= CH_3_	1: 84% (48%)				[88]
			R_2 _= CH_3_	2: 10%				

^I^This isomer was not detected in the product mixture [168];

^II^no oxidation detectable [168];

^III^due to the low enantioselectivities of the reactions, no absolute configuration was assigned [170];

^IV^value not given [170].

In addition to *S. cerevisiae *strains, also alternative yeasts were genetically modified to exhibit beneficial oxidoreductase properties (Table 3 and Additional File 3). *Rhodotorula glutinis *epoxide hydrolase was for example overexpressed in *Pichia pastoris *leading to a 10-fold elevated activity toward (*R*)-styrene oxide conversion compared to the *R. glutinis *wild type. Kinetic resolution of racemic styrene oxide yielded in the (*S*)-styrene oxide with 98% ee and 36% yield [171]. A recombinant *Schizosaccharomyces pombe *strain coexpressing the human cytochrome P450 CYP11B1 mutant V78I and the electron transfer proteins adrenodoxin (Adx) and adrenodoxin reductase (AdR) showed a 2.5-fold higher 11β-hydroxylation activity than the parental strain for the production of hydrocortisone [172]. Furthermore, Peters et al. [173] developed a *Schizosaccharomyces pombe *strain expressing human CYP2D6 and evaluated its principle feasibility for the synthesis of 4'-hydroxymethyl-α-pyrrolidinobutyrophenone (56% yield, 98.5% purity – Table 3 and Additional File 3). Other yeast whole-cell biocatalysts which were engineered to display enzymes on their surface are described in section 3.3.

#### 3.2 Cofactor regeneration using genetically engineered yeasts

As far as organic biotransformations are concerned, the most common application of yeast whole-cell biocatalysts is for asymmetric reductions. For these synthetically useful reactions cofactor-dependent enzymes are required. Considering the cost of NAD(P)^+ ^and NAD(P)H, their stoichiometric application is not economically feasible unless an efficient regeneration method is employed which allows the use of only low amounts [174]. In general, whole-cell biocatalysts provide the cheapest cofactor regeneration system preventing the laborious procedure of enzyme isolation and taking advantage of prolonged enzyme stability and cofactor recycling from cheap auxiliary substrates. However, there exist several disadvantages when employing wild-type whole-cell biocatalysts (see Section 1.1).

Recently, improvements in cofactor regeneration systems were comprehensively reviewed focusing on pyridine nucleotide regeneration [175], cofactor regeneration with genetically engineered bacteria [176] and new methods for the improved regeneration of ATP/NTP, sugar nucleotides and PAPS (3'-phosphoadenosine-5'-phosphosulfate) which is involved in sulfuryl transfer reactions [177]. In most cases however isolated enzymes and genetically modified bacteria were employed. Regarding again *S. cerevisiae*, only traditional approaches were followed, using the yeast for its supply of reducing power and encountering serious drawbacks when trying to freely combine it with desired non-natural substrates. Stewart and coworkers [47,68,168] were among the first scientists who constructed genetically engineered *S. cerevisiae *strains and thereby reduced undesirable dehydrogenase background reactions by deleting single reductase genes. Thus, it became again more attractive to utilize the *S. cerevisiae*-own cofactor regeneration system for the asymmetric reduction of selected ketones. However, considering all attempts it seems that the potential of *S. cerevisiae *as whole-cell redox-biocatalyst has not been fully exploited yet. The huge number of existing *S. cerevisiae *genes encoding for reductases suggests a more complex strategy such as temporarily silencing of metabolic pathways either by reaction engineering as indicated by Kratzer et al. [158] (see Chapter 3.1, Table 3 and Additional File 3) or by regulation of gene expression, a perhaps more challenging approach.

At the same time, metabolic engineers also investigated the impact of cofactor engineering on the redox metabolism of *S. cerevisiae*. Nissen et al. [178] expressed for example the cytoplasmic transhydrogenase from *Azotobacter vinelandii *in *S. cerevisiae *CBS8066. Transhydrogenases, which were not found in yeasts [179,180], catalyze the hydrogen transfer between the two cofactor systems NADH/NAD^+ ^and NADPH/NADP^+^. Nissen's results indicated that when introducing a transhydrogenase in yeast, the conversion of NADP(H) and NAD^+ ^to NADP^+ ^and NADH was favored [178]. This was already observed by Anderlund et al. [181] who constructed recombinant *S. cerevisiae *strains expressing a membrane-bound *E. coli *transhydrogenase. In contrast, Nielsen and coworkers [182] also succeeded in constructing genetically engineered *S. cerevisiae *strains with a modified ammonium assimilation pathway for increased NADPH availability. Therefore, the NADPH-consuming glutamate dehydrogenase (GDH1) was deleted and an alternative pathway consisting of an NADH-consuming GDH2 or the ATP-dependent glutamine synthetase (GLN1) and the NADH-dependent glutamate synthase (GLT1) was introduced. The major redox alteration of the engineered strain was shown by a reduced flux through the pentose phosphate pathway during aerobic growth on glucose. Finally, this switch in cofactor requirement for ammonia assimilation resulted in both an increase in ethanol yield (10%) and a significant reduction of the glycerol yield (~40%) [183]. Furthermore, *S. cerevisiae *strains with a decreased intracellular NADH pool were generated by expressing the *Lactobacillus lactis *H_2_O-forming NADH oxidase [184]. A major consequence of the thus provided NAD^+ ^excess led to a significant reduction in ethanol yield (15%). This strategy could provide a route to reduce and adjust the ethanol content of fermented beverages like beer or wine [184]. The overexpression of alternative oxidases could also result in valuable strains for NAD^+ ^regeneration.

Recently, the methylotrophic yeast *Pichia pastoris *was engineered for efficient NADH regeneration in our laboratory (F. S. Hartner, personal communication). The concept included the decrease in MeOH assimilation by deleting two dihydroxyacetone synthase genes. Thereby, the existing methanol utilization pathway – generating two molecules of NADH while methanol is irreversibly oxidized to CO_2 _– was enhanced. This approach led to improved space-time yields and specific conversion rates for the conversion of acetoin to 2,3-butanediol catalyzed by the overexpressed *S. cerevisiae *butanediol dehydrogenase. In this case, methanol was used as a cosubstrate for NADH regeneration but also as an inducer for the expression of the heterologous catalyst and the endogenous NADH regeneration pathway.

Furthermore, engineered *S. cerevisiae *cells overexpressing hexokinase or glucokinase were reported to almost completely convert adenosine (130 mM) to ATP [185], providing a possible ATP regeneration system [25].

Again *E. coli *and the yeast *S. cerevisiae *are the most often and thoroughly studied organisms regarding attempts to optimize cofactor recycling by genetic engineering. However, especially methylotrophic yeasts provide interesting alternatives for engineering endogenous cofactor recycling pathways.

#### 3.3 Transport limitations and displaying enzymes on the surface of yeast whole-cell biocatalysts

Considering whole-cell processes, it would be ideal if the starting material was transported into the cell and if products were released without any limitation. The production rate would then only be dependent on the cell's metabolic functions including the catalyst's kinetic limitations. In reality, bioprocesses are often drastically limited by barrier functions of the cell wall or membrane. In order to circumvent these limits, permeabilization methods are common practice. Recently, Chen [186] reviewed standard permeabilization protocols including efficient methods for yeasts such as solvent [187-189], detergent [190] or alkaline [191] treatment and air-drying at 42°C [192], respectively. However, classical permeabilization methods often show serious shortcomings causing extensive damage to membrane systems, even cell lysis [193], which makes cofactor regeneration or the reuse of the catalyst impossible. Furthermore, downstream processing can be complicated. Recently, molecular engineering approaches provided an alternative route to solve the cell permeability issue in a way that can be better predicted [186]. Either outer membrane structures were engineered – a method which was up to now only used for bacterial cells – or enzymes were displayed on the cell surface. In the following the later method will be discussed in more detail.

Molecular displaying techniques allow the targeting of heterologous proteins to the surface of yeast strains. They are covalently linked to the internal, skeletal layer of β-1,3- and β-1,6 glucan complexed with chitin and protrude to the outer layer of glycoproteins [194]. This technique provides several advantages: The anchoring to the cell surface can eliminate purification and separation processes and result in increased biocatalyst stability. Benefits of *S. cerevisiae *which has GRAS status and is well suited for the expression of proteins from eukaryotes can be combined with economic aspects like facilitated biocatalyst recycling due to the self-immobilization of the recombinant enzymes, simple cultivation and fast growth to high cell densities. Furthermore, mass transport problems of substrate and/or product across the cell membrane can be prevented as the enzyme, necessary for catalysis, is displayed on the cells' surface [195,196]. This is especially important when polymeric compounds are used as substrates. In spite of these benefits, current disadvantages of whole-cell biocatalysts with enzymes anchored to their cell wall should not be neglected such as current limitations to one- or few-step reactions, the restriction to simple biotransformations independent of costly cofactors and the loss of the protective subcellular environment providing optimal ionic, pH- and redox-conditions for enzymatic reactions. However, one or the other disadvantage could be solved by further genetic engineering approaches such as co-displaying of a cofactor-dependent oxidoreductase and an enzyme for cofactor recycling or the engineering of the outer membrane structure of yeast cells to improve the conditions in the vicinity of the displayed enzyme.

In particular, Japanese scientists succeeded in developing the first enzyme-displaying yeast cells and investigated their application for whole-cell biocatalysis focusing on hydrolases. They studied for example the optical resolution of (*RS*)-1-phenylethanol by enantioselective transesterification with vinyl acetate [197]. Therefore, *S. cerevisiae *MT8-1 cells were employed which displayed the pro-region of *Rhizopus oryzae *lipase (ProROL) by fusing the flocculation functional region of the cell-wall protein Flo1p to the lipase's N-terminus [198]. The conversion resulted in the (*R*)-1-phenylethyl acetate with high yields and enantiomeric excess (> 93%) [197]. Furthermore, Kondo and coworkers [199] applied *S. cerevisiae *cells displaying *Rhizopus oryzae *lipase (ROL) for the optical resolution of (*R,S*)-1-benzyloxy-3-chloro-2-propyl monosuccinate. After a reaction time of 16 h, the ROL-displaying yeast had hydrolyzed the (*R*)-compound with an ee_p_-value of 95.5% and a conversion of 50.2%, whereas the employment of soluble *R. oryzae *lipase (F-AP15, Amano enzyme Inc., Japan) showed quite poor ee and conversion (58.7% ee_p_, 35.9% conv., at 12 h) [199]. The stability of ROL-displaying yeast cells was confirmed by reusing the biocatalyst eight times without significant activity loss. The displaying-technique was also combined with enzyme engineering: Shibamoto et al. [200] constructed a surface-displayed combinatorial library of *R. oryzae *lipase in *S. cerevisiae *which resulted in new biocatalysts with increased activity for the hydrolysis and methanolysis, respectively, of soybean oil. Methanolysis yields in methyl esters constituting a potential biodiesel fuel. Recently, Kaya et al. [201] reported the production of isoflavone aglycones from isoflavone glycosides employing *S. cerevisiae *cells. Three different β-glucosidases from *Aspergillus oryzae *were individually displayed on the yeast cell surface. The engineered yeast strain with the β-glucosidase BGL1 (gene ID: AO090003001511 from the *A. oryzae *RIB40 genome) exhibited the highest β-glucosidase activity and first whole-cell biotransformations were performed to produce the isoflavone aglycones daidzein, glycitein and genistein. Furthermore, Kim et al. [202] described the production of cyclofructans from inulin. They engineered *S. cerevisiae *to display cycloinulooligosaccharide fructanotransferase from *Paenibacillus macerans *on the cell surface. As major product, cycloinulohexaose was detected along with cycloinuloheptaose and cycloinulooctaose as minor products. Yeast surface display was also used by Wittrup and coworkers for the FACS-based selection of horseradish peroxidase variants with enhanced enantioselectivity toward L- and D-tyrosinol, respectively [203].

Another lipase was displayed by Ueda and coworkers [204]. They developed *S. cerevisiae *cells displaying a mutated form of *Candida antarctica *lipase B which was constructed on the basis of the primary sequences of the CALBs from *C. antarctica *CBS 6678 and *C. antarctica *LF 058, respectively. α-Agglutinin was used as anchor protein. The newly generated biocatalyst displayed high thermal stability (*T*_1/2 _(60°C) = 30 min) and > 6-fold higher activity for the hydrolysis of *p*-nitrophenyl butyrate compared to previously reported CALB mutants generated by Zhang et al. (CALB-23G5: 86 nmol/min/μg protein) [205]. However, despite of several advantages such as for example straight-forward library construction and screening, whole cells displaying enzymes still possess their own pool of wild-type enzymes that might act on provided substrates able to diffuse through the cell membrane. Considering especially esterolytic and lipolytic activities which are quite common in all organisms, this might lead to undesirable by-products and a significant reduction of chemical and optical yield.

A great benefit of the enzyme displaying technique is the prevention of mass transport problems of the substrate across the cell membrane. This makes polymeric compounds amenable as substrates for whole-cell biocatalysis. In the following some examples are given describing the successful degradation of different polymeric substrates by recombinant *S. cerevisiae *cells displaying enzymes on their cell surface. More details were summarized by Ueda and Tanaka [206] and Wu et al. [207], respectively. Fukuda et al. [196] produced chitooligosaccharides from a chitosan polymer employing yeast cells with *Paenibacillus fukuinensis *chitosanase on the yeast surface. The products are a remarkable resource for the development of e.g. functional food and diverse materials such as artificial skin [196]. Fujita et al. [208] constructed a *Saccharomyces cerevisiae *whole-cell biocatalyst displaying *Trichoderma reesei *xylanase II for the degradation of xylan. Furthermore, they developed a yeast strain (*S. cerevisiae*) which co-displayed three types of cellulolytic enzymes: *Trichoderma reesei *endoglucanase II, *T. reesei *cellobiohydrolase II and *Aspergillus aculeatus *β-glucosidase 1 [209]. The generated whole-cell biocatalyst performed the saccharification and subsequent fermentation of amorphous cellulose and produced 0.45 g ethanol per gram of carbohydrate consumed (88.5% yield).

Although the cell wall of *Saccharomyces cerevisiae *is the one which is the best characterized among yeasts [210,211], targeting proteins is also applicable to alternative variants. *Kluyveromyces lactis*, for example, was used to test this and cell-associated α-galactosidase activity was detected [194]. Recently, Jiang et al. [212] reported for the first time the displaying of an enzyme on *Pichia pastoris *KM71, namely the lipase LipB52 from *Pseudomonas fluorescens *B52. Engineered *P. pastoris *strains featured a slightly improved thermal stability compared to a *Saccharomyces cerevisiae *EBY100-pLHJ026 strain displaying the same lipase. Furthermore, Yue et al. [213] developed a new plasmid for the display of proteins on *Yarrowia lipolytica *cells providing the basis for applications in the field of whole-cell biocatalysis.

#### 3.4 Synthetic pathways in yeasts

The construction and employment of 'designed strains' for natural product biosynthesis clearly indicate the potential of yeast engineering. In recent years, remarkable progress paved the way for the design of whole-cell biocatalysts capable of producing structurally complex natural products and novel variations thereof. An exponentially increasing number of sequences was elucidated. New bioinformatic tools providing the basis for analyzing this load of information were developed and sophisticated tools for engineering heterologous hosts were established.

Natural products like isoprenoids, flavonoids or polyketides represent structurally complex compounds which are often routinely made in nature but require long and elaborate synthesis routes when classical chemical methods are employed. Compounds of all three classes have attracted attention because of their great commercial potential. The class of isoprenoids includes for example carotenoids which are valuable antioxidants and food and feed additives, steroids which are widely used as drugs constituting anti-inflammatory, contraceptive and anticancer agents, or terpenoids like the well-known diterpenoid taxol with its excellent activity, also against a range of cancers. Flavonoids which derive from the phenylpropanoid pathway possess anti-allergenic, anti-inflammatory, and anti-oxidant activities in humans, and polyketides are applied for the treatment of cancer (adriamycin), cardiovascular diseases (lovastatin), immunosuppression (rapamycin, tacrolimus) or infectious diseases (erythromycin, tetracycline) [214].

As the structural complexity makes the chemical synthesis of natural products quite difficult, fermentation is regarded to be an economically feasible alternative to produce pharmaceutically useful compounds for commercial purposes. Most native organisms producing complex polyketide- or isoprenoid-derived compounds do not provide high-levels of these substances as they tend to grow slowly. In addition, they are often not amenable to genetic manipulation. Thus, alternative heterologous systems with faster growth and established genetic engineering techniques seem to provide an interesting alternative with respect to volumetric productivity. The relatively homogenous processes towards the final products are another great benefit of natural product pathways. Precursors and enzymes of natural product synthesis pathways are closely related and a kind of modular system can be established in order to produce different natural but also novel compounds with therapeutic properties. Improvements of manufacturing processes are thus possible at a more rapid pace. Essential for the understanding of the synthetic pathways are not only genetic techniques for their manipulation but also the molecular understanding of every individual biocatalytic step, hence the information gained by simple one-step biochemical reactions.

So far, *Saccharomyces cerevisiae *represented again an ideal host for synthetic pathway engineering due to its rapid growth, advanced fermentation techniques and well established genetic tools [215]. In the following some examples are given employing *S. cerevisiae*, but also *Candida utilis *which was engineered for carotenoid production (Table 5: Additional File 5 and 6: Additional File 6).

**Table 5:** Synthetic pathways based on isoprenoids, listed in chronological order

**Host organism**	**Engineering steps**	**Substrate**	**Product/Outcome**		**Ref**
*Saccharomyces cerevisiae*	1) Introduction of the *Erwinia uredovora *carotenoid biosynthesis genes [216]*crtE*, *crtB*, *crtI *and *crtY *(→ β-carotene) and *crtE*, *crtB *and *crtI *(→ lycopene), respectively, under the control of *S. cerevisiae *promoters and terminators [221]	galactose	β-Carotene: 0.103 mg/g [CDW]		[221-223]
	2) Introduction of *Erwinia herbicola *carotenoid biosynthesis genes for lycopene, β-carotene and zeaxanthin production under the control of *S. cerevisiae *promoters and terminators [222]		Lycopene: 0.113 mg/g [CDW]		[221-223]
			Zeaxanthin: 0.01% of CDW ~0.2 – 0.05 mg/g [CDW]		[222,223]

*Candida utilis*	1) Introduction of synthetic, codon-optimized *Erwinia uredovora *carotenoid biosynthesis genes (crtE, crtB, crtI and crtY) [216] and *Agrobacterium aurantiacum *carotenoid biosynthesis genes (crtZ and crtW) [217] under the control ot *C. utilis *promoters and terminators:	glucose	Astaxanthin: 0.4 mg/g [CDW]		[219]
	- Astaxanthin: *crtE*, *crtB*, *crtI*, *crtY*, *crtZ *and *crtW*		β-Carotene: 0.4 mg/g [CDW]		[219]
	- β-Carotene: *crtE*, *crtB*, *crtI *and *crtY*				
	- Lycopene: *crtE*, *crtB *and *crtI*				
	2) Improving Lycopene yields [218] by disruption of the *C. utilis *squalene synthase gene (*ERG9*) and overex-pression of the catalytic domain of the *C. utilis *3-hydroxy methylglutaryl CoA reductase gene (*HMG*)		Lycopene: 1.1 mg/g [CDW][219] 7.8 mg/g [CDW][218]		[218,219]

*Saccharomyces cerevisiae *(*fen1*)	1) Transfer of expression cassettes for mature bovine adrenodoxin (ADX), adrenodoxin reductase (ADR), and side chain cleavage cytochrome P450 (P450scc)	galactose		60 mg/L	[241]
	2) Transfer of *Arabidopsis thaliana *Δ7-sterol reductase				
	3) Disruption of Δ22-sterol desaturase (one step of endogenous ergosterol biosynthetic pathway)				

*Saccharomyces cerevisiae *(*fen1*)	1) Transfer of expression cassettes for mature bovine adrenodoxin (ADX), adrenodoxin reductase (ADR), and side chain cleavage cytochrome P450 (P450scc)	galactose		No value given	[241]
	2) Transfer of *Arabidopsis thaliana *Δ7-sterol reductase				
	3) Disruption of Δ22-sterol desaturase (one step of endogenous ergosterol biosynthetic pathway)				
	4) Introduction of type II human 3β-hydroxy-steroid dehydrogenase-isomerase (3β-HSD)				

*Saccharomyces cerevisiae*	1) Rerouting the ergosterol biosynthesis pathway	glucose/ethanol		11.5 mg/L	[228]
	2) Introduction of the mammalian-specific part of the hydrocortisone biosynthetic pathway				
	3) Inactivation of side reactions to steroid biosynthesis dead ends				
	4) Adjusting expression levels for optimized steroid channeling to hydrocortisone				

*Saccharomyces cerevisiae*	1) Introduction of the *Artemisia annua *epicedrol synthase gene	galactose		0.37 mg/L	[229]
	2) Overexpression of a truncated Hydroxy-methylglutaryl CoA reductase (trHmg1p)				
	3) Mutation of the Upc2p transcription factor → introduction of the *upc2-1 *allele with G888D [242]				
	4) Employing the *S.c. *haploid mating type **a**				

*Saccharomyces cerevisiae*	1) Introduction of five Taxol biosynthetic genes from *Taxus *species: geranylgeranyl disphosphate synthase (GGPPS), taxadiene synthase (TS), taxadiene 5α-hydroxylase (THY5a), taxadienol 5α-*O*-acetyl trans-ferase (TAT), taxoid 10β-hydroxylase (THY10b) with necessary modifications for the expression in *S. c*.	simple sugar (glucose, galactose) and [2-^14^C] mevalonic acid for radio-HPLC analysis	taxadien-5α-ol: 0.025 mg/L no taxadien-5α-acetoxy-10β-ol *in vivo*		[230]
	2) Due to restricted THY5a expression, only a very small amount of the intermediate taxadien-5α-ol and no taxadien-5α-acetoxy-10β-ol was detected *in vivo *[230]				

*Saccharomyces cerevisiae*	1) Engineering the farnesyl pyrophosphate (FPP) biosynthetic pathway	simple sugar		~32 mg/L	[226]
	2) Introduction of the *Artemisia annua *L amorphadiene synthase gene (FPP → amorphadiene)				
	3) Cloning the *A. annua *CYP71AV1/CPR (3-step oxidation: amorphadiene → artemisinic acid)				

*Saccharomyces cerevisiae*	1) Follow-up study of [226]:	glucose	~380 mg/L ~120 mg/L		[227]
	2) Engineering the pyruvate dehydrogenase bypass (pyruvate to acetyl-CoA) by overexpression of				
	- *Salmonella *acetyl-CoA synthetase variant (L641P)				
	- *S. cerevisiae *cytosolic acetaldehyde dehydrogenase (*ALD6*)				
	- In strain *S. cerevisiae *EPY224 [226]				
	3) Results: increased levels of mevalonate and amorpha-4,11-diene (~120 mg/L); generally applicable for isoprenoid production				

**Table 6:** Synthetic pathways for polyketide and flavonoid synthesis

**Host organism**	**Engineering steps**	**Substrate**	**Product/Outcome**		**Ref**
*Saccharomyces cerevisiae*	1) Introduction of the *Penicillium patulum *PKS 6-MSAS^I ^gene	YPD (+ glucose)		1.7 g/L (2-fold more than by natural host *Penicillium patulum*)	[232]
	2) Overexpression of the *Bacillus subtilis *surfactin P-pant^II ^transferase (Sfp) gene				

*Saccharomyces cerevisiae*	1) Introduction of the *Penicillium patulum *PKS 6-MSAS^I ^gene	galactose minimal medium		> 200 mg/L for strain expressing the PPTase^II ^from *A. nidulans*	[231]
	2) Introduction of the surfactin P-pant^II ^transferase gene from *Bacillus subtilis *(*sfp*) or from *Aspergillus nidulans *(*npgA*)				

*Saccharomyces cerevisiae *BJ5464 (protease deficient)	1) Introduction of pathways for methylmalonyl-coenzyme A production:	YPD (+ glucose) + propionate and propyl-diketide thioester feed		0.5–1 mg/L with propionyl-CoA-dependent route (PCC-pathway)	[215]
	Propionyl-CoA-dependent route:				
	+ *Salmonella typhimurium *propionyl-CoA synthetase				
	+ *Streptomyces coelicolor *propionyl-CoA carboxylase pathway (PCC)				
	Propionyl-CoA-independent route				
	+ *Streptomyces coelicolor *malonyl/methylmalonyl-CoA ligase pathway (MatB)				
	2) Expression of module 2 from DEBS1^III ^linked to the thioesterase domain (TE) of DEBS3 [243,244]				
	3) Co-expression of five tRNA genes E4, R2, L5, Q2 and P2 [245]				

*Saccharomyces cerevisiae*	1) Introduction of the *Populus trichocarpa *X *Populus deltoides *(= poplar) cDNAs encoding:	selective, synthetic medium supplemented with glucose and galactose, respectively		~3–10 mg/L triple-expressing strains (PAL2/C4H/CPR2 and PAL4/C4H/CPR2, respectively; with slight advantages for the PAL2 expressing strain)	[233]
	+ Phenylalanine ammonia lyase (isoform PAL2 and PAL4, respectively)	feed with [^3^H]phenylalanine [^14^C]cinnamate			
	+ Cinnamate 4-hydroxylase (C4H)				
	+ Cytochrome P450 reductase (CPR2)				

*Saccharomyces cerevisiae*	1) Introduction of the *Helianthus tuberosus *C4H Cytochrome P-450 and *H. tuberosus *NADPH-Cytochrome P-450 reductase	glucose or raffinose addition of L-phenylalanine (1.0 mM)		after 24 h:	[234]
	2) Introduction of the *Rhodotorula glutinis *ATCC 10788 phenylalanine ammonia-lyase (PAL)	induction with galactose		on glucose: 354 μM (→ 58 mg/L)	
				on raffinose: 498 μM (→ 82 mg/L)	

*Saccharomyces cerevisiae *AH22	1) Introduction of the phenylpropanoid pathway:	YPD (+ glucose) and YPL (+ galactose)	*S. cerevisiae *AH22 with PAL, 4CL and CHS produced ~7 mg/L of naringenin and 0.8 mg/L of pinocembrin		[235]
	- *Rhodosporidium toruloides *phenylalanine ammonia lyase (*PAL*)				
	- *Arabidopsis thaliana *4-coumarate:coenzyme A (CoA) ligase (*4CL*)				
	- *Hypericum androsaemum *chalcone synthase (*CHS*)				

*Saccharomyces cerevisiae *YPH499	1) Introduction of the chalcone synthase (CHS) from ripe raspberry (*Rubus idaeus*) or a variant thereof (CHS L214I-F215L)	YPGal-medium (induction with galactose)		raspberry fruit: 1–4 mg/kg	[237]
	2) Introduction of the tobacco 4-coumarate-coenzyme A ligase (4CL)	addition of *p*-coumaric acid (3 mM)		recombinant *E. coli*: 5 mg/L	[236]
				recombinant *S. cerevisiae*: no proper detection of raspberry ketone	[236]

*Saccharomyces cerevisiae *FY23	1) Introduction of the coenzyme-A ligase *4CL216 *gene from hybrid poplar under the control of the yeast ADH2 gene promoter and terminator (→ CAL1) and	SCDL-medium (0.67% yeast nitrogen base, 0.8% glucose and required growth factors) with 10 mg/L *p*-coumaric acid	recombinant *S. cerevisiae*: ~1.5 μg/L		[238]
	2) the resveratrol synthase (VST1) from grapevine (*Vitis vinifera*)				

*Saccharomyces cerevisiae *CEN.PK 113-3b (*ura3 his3*)	1) Introduction of the 4-coumarate: coenzyme A (CoA) ligase (*4CL2 *gene – GenBank accession no. U50846) from *Nicotiana tabacum *cv. Samsun	50 mL yeast nitrogen base medium supplemented with 5 mM *p*-coumaric acid and 2% galactose to induce gene expression		*S. cerevisiae*: ~6 mg/L	[239]
	2) Introduction of the STS gene from *Vitis vinifera *encoding for the stilbene synthase (STS)			recomb. *E. coli *expressing the same enzymes: ~16 mg/L	

^I^PKS = polyketide synthase; 6-MSAS = 6-methylsalicylic acid synthase

^II^PPTase = P-pant transferase = 4'-phophopantetheinyl transferase

^III^DEBS = deoxyerythronolide B synthase from *Saccharopolyspora erythraea*, a typical 'modular' polyketide synthase.

Carotenoids represent one of the first classes of natural compounds produced by recombinant yeast strains. Gene clusters responsible for the synthesis of carotenoids were isolated from carotenogenic bacteria, including for example *Erwinia *sp. and *Agrobacterium aurantiacum*. Their function was elucidated [216,217], and essential genes were introduced to *S. cerevisiae *and *C. utilis*, respectively [218-220]. *S. cerevisiae *and *C. utilis *are capable of accumulating ergosterol as their principle isoprenoid compound. Obviously, this strongly suggested the possibility of redirecting the pathway partly to carotenoid production. Finally, engineered *C. utilis *strains [218,219] surpassed recombinant *S. cerevisiae *strains [221-223] in the production of β-carotene and lycopene (Table 5 and Additional File 5). Actually, *C. utilis *strains performed even better than engineered *E. coli*, especially for lycopene production (*C. utilis*: up to 7.8 mg/g CDW [218]). Engineered *E. coli *strains were found to accumulate more than 1 mg/g CDW of lycopene, β-carotene and zeaxanthin, and more than 0.5 mg/g CDW of astaxanthin [220].

**Figure 3:**
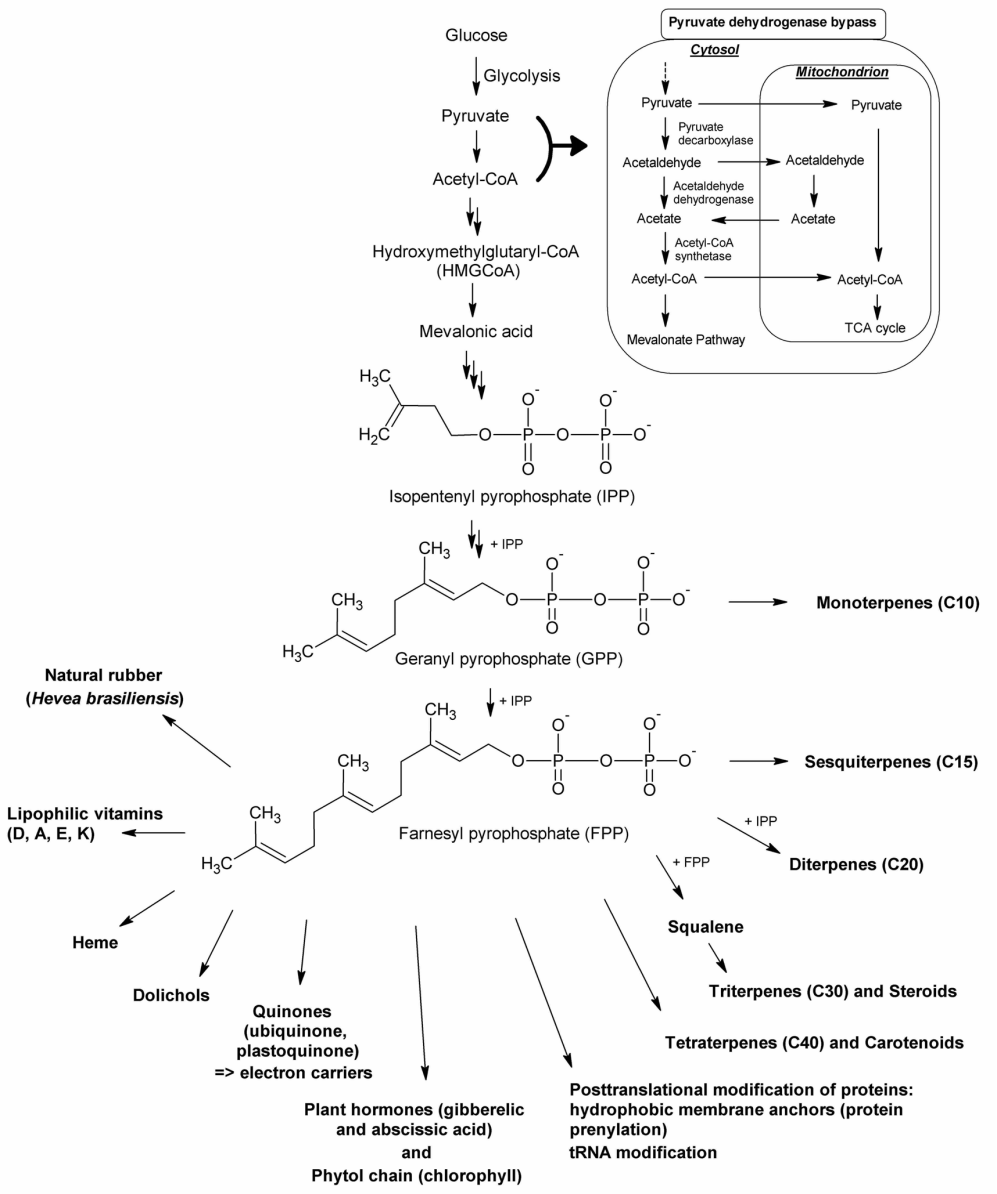
Overview of the biosynthetic pathway of farnesyl pyrophosphate – a pivotal intermediate for several essential pathways and various products [224]. Included is a schematic representation of the pyruvate dehydrogenase bypass whose engineering was shown to be effective for high-level isoprenoid production in *S. cerevisiae *[227].

In general, the pivotal intermediate of all isoprenoid compounds produced in a cell is farnesyl pyrophosphate [224], which also represents the branching point of the diverse isoprenoid biosynthetic pathways (Figure 3). Eukaryotic cells possess a variety of isoprenoid compounds, which are involved in a vast array of cellular processes such as post-translational modification of proteins (prenylation) and tRNA modification. They represent compounds of the lipid bilayer, electron transporters during respiration, cofactors involved in enzyme catalysis, and hormones for signal transduction.

In 1999, Dimster-Denk et al. [225] investigated the regulation of the isoprenoid pathway in *S. cerevisiae*. They evaluated the expression of all genes fusing them to reporter constructs which allowed both, the profiling of gene transcription and translation. Therefore, the Acacia's yeast Genome Reporter Matrix™ (GRM) was used, consisting of reporter-gene-fusion-constructs to > 95% of all *S. cerevisiae *genes encoding proteins.

A recent highlight of synthetic pathway engineering was reported by Keasling and coworkers [226]. They engineered *Saccharomyces cerevisiae *for artemisinic acid (a structurally complex precursor of artemisinin) production with titers of up to 100 mg/L. As natural artemisinin – extracted from the plant *Artemisia annua *L – is highly effective against parasitic *Plasmodium *spp., its improved production would preserve the drug's short supply and make it more affordable to most malaria sufferers. Therefore, Ro et al. [226] improved the mevalonate pathway, introduced amorphadiene synthase, and a novel cytochrome P450 monooxygenase (CYP71AV1) from *A. annua *that performed together with its native redox partner CPR (NADPH:cytochrome P450 oxidoreductase) from *A. annua *a three-step oxidation of amorpha-4,11-diene to artemisinic acid. The synthesized artemisinic acid was then transported out of the cell and was retained on the outside of the engineered yeast, constituting a simple and inexpensive purification process for product isolation. In a next step, Shiba et al. [227] enhanced the supply of acetyl-CoA to the mevalonate pathway and gained elevated levels of amorphadiene. This was achieved by engineering the pyruvate dehydrogenase bypass in *S. cerevisiae*. Overproduction of acetaldehyde dehydrogenase and introduction of a *Salmonella enterica *acetyl-CoA synthetase variant increased the carbon flux into the mevalonate pathway. This engineering step is generally applicable to the improvement of the production of isoprenoid compounds in yeast (see also Figure 3 and last entry in Table 5 and Additional File 5).

Furthermore, the major adrenal glucocorticoid of mammals, hydrocortisone, was artificially synthesized by recombinant *Saccharomyces cerevisiae *[228]. Therefore, a fully self-sufficient biosynthetic pathway involving 13 engineered genes was assembled and expressed in a single yeast strain. Endogenous sterol biosynthesis was rerouted to produce compatible sterols which served as substrates for the heterologous part of the pathway. The biosynthesis involved eight mammalian proteins and further optimization required the modulation of two mitochondrial systems and the disruption of unwanted side reactions associated with the formation of several unnecessary gene products. Jackson et al. [229] succeeded in engineering *S. cerevisiae *for the increased production of the yeast-foreign sesquiterpene epi-cedrol. Yields of up to 0.37 mg/L were obtained after introduction of the *Artemisia annua *epi-cedrol synthase gene, overexpression of a truncated hydroxy-methylglutaryl CoA reductase and use of a *S. cerevisiae upc2-1 *mating type **a **background. Finally, the first steps of the Taxol biosynthesis pathway were transferred to *S. cerevisiae*. However, DeJong et al. [230] determined only small amounts (0.025 mg/L) of taxadien-5α-ol but no *in vivo *detection of taxadien-5α-acetoxy-10β-ol was possible.

The production of polyketide natural products in heterologous hosts, incapable of making polyketides on their own, requires first of all the introduction of a functional polyketide synthase (PKS). Similar to fatty acid biosynthesis, PKS is responsible for the condensation of acetyl-CoA and malonyl-CoA moieties [231]. Kealey et al. [232] and Wattanachaisaereekul et al. [231] engineered *S. cerevisiae *for the heterologous production of 6-methylsalicylic acid (1.7 and 0.2 g/L, respectively). Mutka et al. [215] first introduced pathways for the production of methylmalonyl-coenzyme A which constitutes a precursor for complex polyketides. Therefore two different routes were chosen, namely a propionyl-CoA-dependent and a propionyl-CoA-independent one. Furthermore, they achieved to demonstrate that the methylmalonyl-CoA, which was produced by the yeast strain, is further converted to a triketide lactone (Table 6 and Additional File 6, Entry 3). 0.5 to 1 mg/L of the triketide lactone were obtained with the propionyl-CoA-dependent route.

Ro et al. [233] engineered *S. cerevisiae *in order to reconstitute the entry point of phenylpropanoid metabolism which should enable higher yields of flavonoids from yeast. Therefore, the *Populus *sp. enzymes phenylalanine ammonia lyase (isoform PAL2 and PAL4, respectively), cinnamate 4-hydroxylase (C4H) and the cytochrome P450 reductase (CPR2) were introduced in the yeast strain. At the end, ~3–10 mg/L of *p*-coumarate were obtained employing the triple-expressing strains. Lately, Vannelli et al. [234] reached 498 μM (= 82 mg/L) of *trans-p*-hydroxycinnamic acid (= *p*-coumarate) with recombinant *S. cerevisiae *cells expressing the plant C4H Cytochrome P-450 and Cytochrome P-450 reductase – from *Helianthus tuberosus *– and the *Rhodotorula glutinis *phenylalanine ammonia lyase. The highest levels of *p*-coumarate were achieved in cultures growing on raffinose and supplemented with 1.0 mM of L-phenylalanine.

Jiang et al. [235] employed *S. cerevisiae *for the biosynthesis of naringenin, the central precursor of various flavonoids. This was accomplished by the recombinant expression of one yeast and two plant enzymes, namely phenylalanine ammonia lyase from *Rhodosporidium toruloides*, 4-coumarate:coenzyme A ligase from *Arabidopsis thaliana *and chalcone synthase from *Hypericum androsaemum*. They obtained ~7 mg/L of naringenin and 0.8 mg/L of pinocembrin (Table 6 and Additional File 6, Entry 6). Beekwilder et al. [236] cloned plant genes involved in the biosynthesis of raspberry ketone [4-(4-hydroxyphenyl)-butan-2-one] and thus constructed microbial strains for the synthesis of one of the most expensive flavor components. In this case, the recombinant yeast strain converted most of the precursor (*p*-coumaric acid) into hydroxyphenyl-propionic acid and this hampered the proper detection of raspberry ketone by GC-MS. However, they achieved to construct a recombinant *E. coli *BL21 strain expressing tobacco 4CL2 (4-coumarate-coenzyme A ligase) and raspberry CHS (chalcone synthase from *Rubus idaeus*) which yielded in 5 mg/L of raspberry ketone. For this purpose, it was grown in a fermenter for 46 h and enzyme production was induced by the addition of IPTG and *p*-coumaric acid. In nature, raspberry ketone occurs in concentrations between 1 and 4 mg/kg raspberries [237].

Several groups also engineered *Saccharomyces cerevisiae *for the synthesis of the antioxidant resveratrol which can be found especially in red wine. In 2000, González-Candelas et al. [161] already generated a recombinant yeast strain expressing the *Candida molischiana bglN *gene encoding for a β-glucosidase in the industrial wine yeast *S. cerevisiae *T_73 _(CECT1894). In wines produced by the transgenic yeast strain, the content of *trans*- and *cis*-resveratrol, respectively, was elevated to ≥ 0.75 μM compared to ≤ 0.25 μM in wines made with the wild-type. Then, Becker et al. [238] reported for the first time the reconstruction of a biochemical pathway for resveratrol biosynthesis in *S. cerevisiae *cells. However, only low levels of resveratrol (~1.5 μg/L) resulted employing a recombinant *S. cerevisiae *strain co-expressing the coenzyme-A ligase from a hybrid poplar and the grapevine resveratrol synthase gene (Table 6 and Additional File 6, Entry 8). In 2006, Beekwilder et al. [239] succeeded in producing significantly higher amounts of resveratrol with engineered yeast strains (~6 mg/L). They introduced the 4-coumarate:coenzyme A ligase (4CL) from *Nicotiana tabacum *cv. Samsun and the stilbene synthase (STS) from *Vitis vinifera *into *S. cerevisiae *CEN.PK 113-3b. Using the same biosynthetic genes, the recombinant yeast production strain was not much less efficient compared to an *E. coli *production strain (~16 mg/L). Regarding the application in human nutrition, the food-grade status of yeast is a great advantage over *E. coli*.

Finally, Branduardi et al. [240] engineered *S. cerevisiae *for the biosynthesis of L-ascorbic acid (= vitamin C) starting from D-glucose. They cloned and expressed five different genes under the control of the *S. cerevisiae *TPI promoter, namely the gene encoding for the *Arabidopsis thaliana *mannose epimerase, the gene for the *A. thaliana *myo-inositol phosphatase/L-galactose-1-P-phosphatase, the L-fucose guanylyl-transferase from *Rattus norvegicus*, the *A. thaliana *L-galactose dehydrogenase and the *S. cerevisiae *D-arabinono-1,4-lactone oxidase in order to prevent a bottleneck at the end of the pathway. The best conversion yielded in > 0.1 mg/L/OD L-ascorbic acid. In addition, they found out that intracellular accumulation of L-ascorbic acid led to an improved robustness of recombinant yeast strains towards different stress conditions, such as oxidative stress or the presence of organic and inorganic acids, respectively [240].

On the one hand, the above summarized attempts to reconstruct and engineer synthetic pathways in yeasts clearly depict the potential for the creation of future yeast whole-cell factories. The huge wealth of basic knowledge gained in the course of this work has already improved the understanding of more and more subcellular metabolic networks, their regulation and possible modulation. On the other hand, low yields reported for almost all complex products indicate that still major limitations exist which prevent todays yeast cell factories to be directly put to application. Examples with a high potential for an implementation in near future might be a further developed designer strain for the production of artemisinic acid or resveratrol. Especially for the applications in human nutrition, several yeast strains will provide interesting alternatives due to their food-grade status.

## Conclusion

Without any doubt, *Saccharomyces cerevisiae *is the most thoroughly investigated eukaryotic microorganism and therefore the most frequently employed yeast strain in yeast-mediated whole-cell biotransformations. Regarding classical approaches, *S. cerevisiae *was almost always among the first biocatalysts chosen to solve the corresponding catalytic problem. However, finally only three types of enzymatic reactions were successfully performed with *S. cerevisiae *whole cells, namely oxidoreductase- (E.C. 1.-.-.-), hydrolase- (E.C. 3.-.-.-) and lyase- (E.C. 4.-.-.-) mediated biotransformations. The same is true for alternative yeast strains which were found due to biodiversity screening approaches. Alternative yeast strains employed for classical whole-cell biocatalysis are for example *Candida *sp., *Cryptococcu*s sp., *Geotrichum *sp., *Issatchenkia *sp., *Kloeckera *sp., *Kluyveromyces lactis*, *Pichia *sp. (including *Hansenula polymorpha *= *P. angusta*), *Rhodotorula *sp., *Rhodosporidium *sp., *Schizosaccharomyces pombe*, *Torulopsis *sp., *Trichosporon *sp., *Yarrowia lipolytica *and *Zygosaccharomyces rouxii*.

Yeast strains employed for biotransformations in industry include *Candida *sp., *Cryptococcus laurentii*, *Geotrichum candidum*, *Pichia *sp., *Rhodotorula rubra*, *Saccharomyces cerevisiae*, *Trigonopsis variabilis*, and *Zygosaccharomyces rouxii*.

Up to now only wild-type yeast strains were employed for industrial applications except for *Pichia pastoris *strains expressing the phenylalanine dehydrogenase from *Thermoactinomyces intermedius *for the production of a chiral building block for Omapatrilat synthesis. Again, most of the industrial biotransformations catalyzed by yeast whole cells were oxidation or reduction reactions. However, three lyase-based reactions were encountered: *S. cerevisiae *for the production of (1*R*)-phenylacetylcarbinol; *Rhodotorula rubra *for the production of L-phenylalanine and *Candida rugosa *for (*R*)-β-hydroxy-n-butyric and (*R*)-β-hydroxy-isobutyric acid production.

Regarding engineered yeast whole-cell biocatalysts, again, oxidoreductase reactions are favored. This also includes the engineering of more efficient cofactor regeneration systems whereas yeast surface display approaches focus on hydrolytic enzymes.

For every biocatalyst engineer, the construction and employment of synthetic pathways pose probably the greatest challenge as they require the interrelation of diverse fields of research. However, they also elucidate and indicate the catalytic potential and limits of yeast strains and the capability of metabolic engineering including available genetic tools. In future, investigations in all different fields related to whole-cell biocatalysis are required in order to expand the scope of valuable biocatalytic reactions.

## Competing interests

The authors declare that they have no competing interests.

## Authors' contributions

Both authors suggested the topic of this review. BP drafted the manuscript. AG revised it critically and gave final approval of the version to be published. All authors read and approved the final manuscript.

## Supplementary Material

Additional file 1 **Table 1**. Click here for file

Additional file 2 **Table 2**. Click here for file

Additional file 3 **Table 3**. Click here for file

Additional file 4 **Table 4**. Click here for file

Additional file 5 **Table 5**. Click here for file

Additional file 6 **Table 6**. Click here for file
