# Supplementary material for: Yeast cell factories for fine chemical and API production
Source: Microb Cell Fact. 2008 Aug 7;7:25. doi: 10.1186/1475-2859-7-25 (PMC2628649; doi:10.1186/1475-2859-7-25)
Supplement: Additional file 1 — Table 1. [file 1475-2859-7-25-S1.doc]

## Table 1: Examples of wild-type yeast whole-cell biocatalysts for the reduction of C=O bonds and comparison to *S. cerevisiae*, if provided.

| **Whole-Cell Biocatalyst** | **Substrate** | **Product** | **Performance: yield (ee)** | **Ref.** |
| --- | --- | --- | --- | --- |
| *Candida parapsilosis*  IFO 1396 |  |  | *C.p.*: 60% (98% *S*) | [61] |
| *Candida arborea*  IAM 4147 |  |  | *C.a.*: 37% (99% *R*) |  |
| *Issatchenkia scutulata*  IFO 10070 |  |  | *I.s.*: 48% (99% *R*) |  |
| *Kluyveromyces lactis*  IFO 1267 |  |  | *K.l.*: 99% (93% *R*) |  |
| *Candida tropicalis*  PBR-2 MTCC 5158 |  |  | *C.t.*: >84% conv.^a^ (>99% *S*) | [62] |
| *Geotrichum candidum*  CBS 233.76  with Amberlite XAD-1180 |  |  | *G.c.*: 95% (>98% *S*) | [60] |
| *Rhodotorula mucillaginosa*  CBS 2378 |  |  | *R.m.*: 88% (>99% *R*) |  |
| *Saccharomyces cerevisiae*^b^ |  |  | *S.c.*: 95% (41% *R*) |  |
| *Saccharomyces montanus* CBS 6772 |  |  | *S.m.*: ee_cis_ = 93% (1*S*,2*S*) cis:trans = 96:4 | [57] |
| *Saccharomyces cerevisiae* (Type II from Sigma) |  |  | *S.c.*: ee_cis_ = 90% (1*S*,2*S*)  cis:trans = 95:5 |  |
| *Saccharomyces cerevisiae* (dried baker’s yeast) |  |  | *S.c.*: - (94% *R*) | [58] |
| *Saccharomyces cerevisiae* |  |  | *S.c.*: 75% (>99%);  96% de | [59] |
| *Pichia mexicana*  CECT^c^ 11015 |  |  | *P.m.*: 85-86% (95% *R*) | [56] |
| *Saccharomyces cerevisiae*  CECT^c^ 1317 |  |  | *S.c.*: 48% (75% *R*) |  |
| *Yarrowia lipolytica*  CECT^c^ 1240 |  |  | *Y.l.*: 87-88% (99% *S*) |  |
| *Saccharomyces cerevisiae*  (Type II, Sigma) |  |  | *S.c.*: 32% (83% *S*) |  |
| *Kluyveromyces marxianus* CBS 600  *Saccharomyces cerevisiae* GIV 2009^d^ |  |  | *K.m.*:  **2-PE:**  26.5 g L^-1^ in org. phase^e^  STY:^f^ 0.33 g L^-1^ h^-1^  **2-PEAc:**  6.1 g L^-1^ in org. phase^e^  STY:^f^ 0.08 g L^-1^ h^-1^  *S.c.*:  **2-PE:**  24.0 g L^-1^ in org. phase^g^  after 166 h  STY:^f^ 0.14 g L^-1^ h^-1^ | [63]  [64] |

^a^conv. = conversion; ^b^baker’s yeast from Distillerie Italiane, Eridania group; ^c^Spanish type culture collection CECT, Valencia; ^d^The wild type strain *S. cerevisiae* Giv 2009 was from Givaudan Ltd. (Dübendorf, Switzerland);

^e^organic phase = Polypropylene glycol 1200 [63]; ^f^STY = space-time yield; calculated from *t* = 0 until *t* where the maximum product concentration was reached; ^g^organic phase = oleic acid [64].
