# Supplementary material for: Yeast cell factories for fine chemical and API production
Source: Microb Cell Fact. 2008 Aug 7;7:25. doi: 10.1186/1475-2859-7-25 (PMC2628649; doi:10.1186/1475-2859-7-25)
Supplement: Additional file 2 — Table 2. [file 1475-2859-7-25-S2.doc]

## Table 2: Industrial biotransformations [74] employing wild type yeast whole-cell biocatalysts ordered by E.C. numbers

| **Yeast strain** | **Enzyme name**  **(E.C. No.)** | **Reaction** | **Product / *Application*** | **Company** |
| --- | --- | --- | --- | --- |
| *Zygosaccharomyces rouxii* | Alcohol NAD^+^ oxidoreductase (1.1.1.1) |  | LY 300164 = Benzodiazepine /  ***tested for treating amyotrophic lateral sclerosis*** | Eli Lilly and Company, USA |
| *Geotrichum candidum* | Dehydrogenase, NADPH-dependent (1.1.X.X) |  | chiral β-hydroxy ester / ***precursor for cholesterol antagonist inhibiting HMG-CoA reductase*** | Bristol-Myers Squibb, USA |
| *Candida sorbophila* | Dehydrogenase (1.1.X.X) |  | (*R*)-amino alcohol / ***intermediate for a β-3-agonist used for obesity therapy, and to decrease hy-pertension, coronary artery dis-ease and the level of associated type II diabetes*** | Merck & Co, Inc., USA |
| *Pichia methanolica* | Reductase (1.1.X.X) |  | Ethyl-5-(*S*)-hydroxyhexanoate and 5-(*S*)-hydroxyhexanenitrile /  ***chiral building blocks*** | Bristol-Myers Squibb, USA |
| *Candida boidinii* (**E2**) [and *E. coli* with cloned **E1**]^a^ or  *Pichia pastoris* (**E2** + cloned **E1**)^b^ | **E2**: FDH (1.2.1.2)  [**E1**: PheDH (1.4.1.20) from *Thermoactino-myces intermedius*] | 2 | (*S*)-2-Amino-5-(1,3-dioxolan-2-yl)-pentanoic acid / ***one building block for Omapatrilat synthesis (ACE and NEP inhibitor)***^c^ | Bristol-Myers Squibb, USA |
| *Trigonopsis variabilis* ATCC 10679 | D-Amino acid oxidase  (1.4.3.3) |  | L-6-Hydroxynorleucine /  ***chiral intermediate for synthesis of*** ***vasopeptidase inhibitor and other antihypertensive metalloproteinase inhibitors*** | Bristol-Myers Squibb, USA |
| Baker‘s yeast  (=*Saccharomyces cerevisiae*) | Reductase (1.X.X.X) |  | ***The corresponding dione is an intermediate for the synthesis of natural 3-hydroxycarotenoids (e.g. cryptoxanthin, zeaxanthin) and other terpenoid compounds.*** | Hoffmann La-Roche, CH |
| *Cryptococcus laurentii* (**E1**)  [and *Achromobacter obae* (**E2**)]^a^ | **E1**: Lactamase (3.5.2.11) and  [**E2**: Racemase (5.1.1.15)]^a^ |  | L-Lysine /  ***nutrient and food supplement*** | Toray Industries Inc., Japan |
| *Saccharomyces cerevisiae* | Pyruvate decarboxylase (4.1.1.1) |  | PAC 🡪 ephedrine and pseudoephedrine /  ***treatment of asthma, hay fever, and used for bronchodilating agent and decongestant*** | Krebs Biochemicals & Industries Ltd., India |
| *Candida rugosa* | Enoyl-CoA hydratase (4.2.1.17) |  | (*R*)-β-Hydroxy-n-butyric acid and (*R*)-β-Hydroxy-isobutyric acid /  ***chiral synthons for carbapenem intermediate and captopril (ACE-inhibitor)^c^, respectively*** | Kanegafuchi Chemical Industries Co., Ltd., Japan |
| *Rhodotorula rubra* | L-Phenylalanine ammonia-lyase (4.3.1.5) |  | L-phenylalanine / ***artificial sweetener aspartame, parenteral nutrition, chiral building block for Rutamycin B synthesis*** | Genex Corporation, USA |

^a^Bacterial whole-cell biocatalyst. ^b^In this case, also engineered *P. pastoris* cells were employed. ^c^ACE = angiotensin-converting enzyme; NEP = neutral endopeptidase.
