# Supplementary material for: Yeast cell factories for fine chemical and API production
Source: Microb Cell Fact. 2008 Aug 7;7:25. doi: 10.1186/1475-2859-7-25 (PMC2628649; doi:10.1186/1475-2859-7-25)
Supplement: Additional file 3 — Table 3. [file 1475-2859-7-25-S3.doc]

## Table 3: Examples for one- to two step enzymatic reactions employing engineered yeast whole-cell biocatalysts (+ overexpression; - knockout/deletion)

| **Whole-Cell Biocatalyst** | **Engineering**  **+ overexpression**  **- deletion** | **Substrate** | **Product** | **Performance: yield (ee)** | | **Ref.** |
| --- | --- | --- | --- | --- | --- | --- |
| *Saccharomyces cerevisiae* | - β-keto reductase |  |  | 53% (90%)  (slow, continuous substrate feed) | | [151] |
| *Saccharomyces cerevisiae* | A: +Fasp, -Gre2p, -Ypr1p^IV^  B: -Fasp, +Gre2p^IV^ | a: R_1_ = Me; R_3_ = Me  b: R_1_ = Et; R_3_ = Me  c: R_1_ = Me; R_3_ = Et  d: R_1_ = Et; R_3_ = Et  e: R_1_ = *n*-Pr; R_3_ = Et |  | A: | d: 78% (91% *R*)^II^  e: 26% (>98% *RI*)^I^ | [152] |
|  |  |  |  | B: | a: 76% (>98% *S*)^I^  b: 85% (>98% *S*)^I^  c: 83% (>98% *S*)^I^  d: 87% (>98% *S*)^I^  e: 90% (>98% *S*)^I^ |  |
| *Saccharomyces cerevisiae* | A: - Gre2p, + Ypr1p^IV^  B: + Gre2P, - Ypr1p^IV^ | a: R_2_ = Me  b: R_2_ = Et  c: R_2_ = allyl  d: R_2_ = propargyl |  | A: | a: 90% (98% *syn*)^II^  b: 89% (83% *syn*)^II^  c: 92% (65% *syn*)^II^  d: 75% (>98% *syn*)^II^ | [152] |
|  |  |  |  | B: | a: 86% (70% *syn*)^I^  b: 73% (67% *anti*)^I^  c: 67% (>98% *anti*)^I^  d: 70% (>98% *anti*)^I^ |  |
| *Saccharomyces cerevisiae* | A: +Ymr226c^III^  B: +Ara1p^III^  C: +Ypr1p^III^ | R_1_ = Me; R_2_ = Me |  | A: 72% (87%, *S*)^I^  B: 48% (91%, *S*)^I^  C: 62% (87%, *S*)^I^ | | [153,154] |
| *Saccharomyces cerevisiae* | + reductase gene YDR368w^V^ | bicyclo[2.2.2]octane-2,6-dione | (1*R*,4*S*,6*S*)-6-hydroxy-bicyclo[2.2.2]octane-2-ol | 97% de  >99% ee  84% yield | | [155] |
| *Saccharomyces cerevisiae* | + multiple copies of  *Candida tenuis* xylose reductase variant W23F | a: R = H  b: R = Ph  c: R = *o*-Cl-Ph  d: R = *m*-Cl-Ph  e: R = *p*-Cl-Ph  f: R = *p-*CN-Ph |  | a: 35% (>99.9%, *S*)^VI^  b: 80% (>99.9%, *R*)^VI^  c: 76% (99.6%, *R*)^VI^  d: 14% (>99.9%, *R*)^VI^  e: 74% (99.9%, *R*)^VI^  f: 100% (99.4%, n.d.)^VI^ | | [158] |
| *Saccharomyces cerevisiae* | + *Antirrhinum majus* benzoic acid methyltransferase (BAMT) |  |  | ~1 mg methyl benzoate per L of culture (with OD_600_ = 1), after 24 h | | [160] |
| *Pichia pastoris* | + *Rhodotorula glutinis* epoxide hydrolase |  |  | ~ 36% (>98%)  (theoretical yield: 50%) | | [171] |
| *Schizosaccharo-myces pombe* | + human CYP11B1-V78I  + electron transfer proteins adrenodoxin (Adx) & adre-nodoxin reductase (AdR) |  |  | 2.5-fold higher activity compared with parental strain | | [172] |
| *Schizosaccharo-myces pombe* | + human CYP2D6 |  |  | 56% yield  98.5% purity | | [173] |
| *Schizosaccharo-myces pombe* | + *Trigonopsis variabilis* D-Amino Acid Oxidase (multi copies)  - catalase | Cephalosporin C | α-ketoadipyl-7-cephalosporanic acid and  glutaryl-7-amino-cephalosporanic acid | 550 U/g CDW  increased mechanical resistance | | [191] |

^I^carbon source: glucose

^II^carbon source: galactose

^III^Ara1p = *S. cerevisiae* reductase, NADPH-dependent; Ypr1p = *S. cerevisiae* reductase, NADPH-dependent; YMR226c = *S. cerevisiae* short chain dehydrogenase ORF [153].

^IV^Fasp = *S. cerevisiae* fatty acid synthase; Gre2p = *S. cerevisiae* α-acetoxy ketone reductase; Ypr1p = *S. cerevisiae* aldo-keto reductase [152].

^V^*S. cerevisiae* 2-methylbutyraldehyde reductase (NCBI accession number: NP 010656)

^VI^Whole-cell bioreductions of α-keto esters (10 mM) under anaerobic conditions using 1 M ethanol as co-substrate; n.d. = not determined.
