# Supplementary material for: Yeast cell factories for fine chemical and API production
Source: Microb Cell Fact. 2008 Aug 7;7:25. doi: 10.1186/1475-2859-7-25 (PMC2628649; doi:10.1186/1475-2859-7-25)
Supplement: Additional file 4 — Table 4. [file 1475-2859-7-25-S4.doc]

## Table 4: Examples for one step enzymatic reactions employing the designer yeast made by Stewart and coworkers [88,167-170], namely *Saccharomyces cerevisiae* overexpressing the *Acinetobacter* sp. NCIB 9871 cyclohexanone monooxygenase. The data is chronologically ordered.

| **Entry** | **Substrate** | **Product** | **Substrate / Product Code** | **Performance: yield (ee)** | | | | | | | | | | **Ref.** |
| --- | --- | --- | --- | --- | --- | --- | --- | --- | --- | --- | --- | --- | --- | --- |
| 1 |  |  | a: R = Me  b: R = Et  c: R = Pr^i^  d: R = Pr  e: R = allyl | a: 83% (≥98%)  b: 74% (≥98%)  c: 60% (≥98%)  d: 63% (92%)  e: 62% (95%) | | | | | | | | | | [167,168] |
| 2 |  |  | a: R = Me  b: R = Et  c: R = Pr^i^  d: R = Pr  e: R = allyl  f. R = n-butyl |  | | | 1 | | | 2 | | | | [168,169] |
|  |  |  |  | a: | | | 50% (49%) | | | ---^I^ | | | |  |
|  |  |  |  | b: | | | 79% (95%) | | | 69% (≥98%) | | | |  |
|  |  |  |  | c: | | | 41% (≥98%) | | | 46% (96%) | | | |  |
|  |  |  |  | d: | | | 54% (97%) | | | 66% (92%) | | | |  |
|  |  |  |  | e: | | | 59% (≥98%) | | | 58% (≥98%) | | | |  |
|  |  |  |  | f: | | | 59% (≥98%) | | | 64% (98%) | | | |  |
| 3 |  |  | a: R = Me  b: R = Et  c: R = Pr^i^  d: R = Pr  e: R = allyl  f. R = n-butyl |  | | 1 | | | 2 | | | | 3 | [168] |
|  |  |  |  | a: | | 71% ( % y l- T20000000000000000000000000000000000000000000000000000000000000000000000000000000000000000000000000000000000≥98%) | | | 60% (≥98%) | | | | ---^I^ |  |
|  |  |  |  | b: | | 18% (70%) | | | 20% (70%) | | | | ---^I^ |  |
|  |  |  |  | c: | | N.R.^II^ | | | ---^I^ | | | | N.R.^II^ |  |
|  |  |  |  | d: | | 11% (≥98%) | | | ---^I^ | | | | 8% (83%) |  |
|  |  |  |  | e: | | 15% (97%) | | | ---^I^ | | | | 9.3% (≥98%) |  |
|  |  |  |  | f: | | 37% (56%) | | | ---^I^ | | | | 11% (84%) |  |
| 4 |  |  | a: R = n-Bu  b: R = n-Hex  c: R = n-Oct  d: R = n-C_11_H_23_ |  | | | 1 | | | | 2 | | | [170] |
|  |  |  |  | a: | | | 18% (≥98%) | | | | 32% (≥98%) | | |  |
|  |  |  |  | b: | | | 32% (≥98%) | | | | 42% (≥98%) | | |  |
|  |  |  |  | c: | | | 25% (≥98%) | | | | 14% (≥98%) | | |  |
|  |  |  |  | d: | | | 39% (≥98%) | | | | 37% (≥98%) | | |  |
| 5 |  |  | a: R = n-Pr  b: R = n-Hex |  | 1^III^ | | | 2^III^ | | | | 3^III^ | | [170] |
|  |  |  |  | a: | 27% (13%) | | | -^IV^ (33%) | | | | -^IV^ (60%) | |  |
|  |  |  |  |  | overall yield: 44%; ratio 2 : 3 = 83 : 17 | | | | | | | | |  |
|  |  |  |  | b: | 54% (29%) | | | (60%) | | | | -^IV^ | |  |
|  |  |  |  |  | overall yield: 20%; ratio 2 : 3 = >99 : <1 | | | | | | | | |  |
| 6 |  | +  + | a: R = Ph  b: R = Bu^t^  c: R = Bu^n^ | a: 95% (>99%)  b: 47% (99%)  c: 53% (74%) | | | | | | | | | | [88] |
| 7 |  | + | a: R_1_ = H  R_2_ = H  b: R_1_ = H  R_2_ = Ph | 1a: 18% (90%) (2a: 19% yield)  1b: 30% (30%) (no sulfone 2b detected) | | | | | | | | | | [88] |
| 8 |  | +  1 a-c 2 a-b | a: R_1_ = H  R_2_ = H  b: R_1_ = H  R_2_ = CH_3_  c: R_1_ = H  R_2_ = Ph | 1a: 20% (75%) [2a: 45% yield]  1b: 16% (20%) [2b: 15% yield 76% ee]  1c: 74% (20%) [no sulfone 2c detected] | | | | | | | | | | [88] |
| 9 |  | +  1 2 | R_1_ = CH_3_  R_2_ = CH_3_ | 1: 84% (48%)  2: 10% | | | | | | | | | | [88] |

^I^This isomer was not detected in the product mixture [168];

^II^no oxidation detectable [168];

^III^due to the low enantioselectivities of the reactions, no absolute configuration was assigned [170];

^IV^value not given [170].
