# Supplementary material for: Yeast cell factories for fine chemical and API production
Source: Microb Cell Fact. 2008 Aug 7;7:25. doi: 10.1186/1475-2859-7-25 (PMC2628649; doi:10.1186/1475-2859-7-25)
Supplement: Additional file 5 — Table 5. [file 1475-2859-7-25-S5.doc]

Table 5: Synthetic pathways based on isoprenoids, listed in chronological order

| **Host organism** | **Engineering steps** | **Substrate** | **Product / Outcome** | **Ref** |
| --- | --- | --- | --- | --- |
| *Saccharomyces cerevisiae* | 1. Introduction of the *Erwinia uredovora* carotenoid biosynthesis genes [216] *crtE*, *crtB*, *crtI* and *crtY* (🡪 β-carotene) and *crtE*, *crtB* and *crtI* (🡪 lycopene), respectively, under the control of *S. cerevisiae* promoters and terminators [221] 2. Introduction of *Erwinia herbicola* carotenoid biosynthesis genes for lycopene, β-carotene and zeaxanthin production under the control of *S. cerevisiae* promoters and terminators [222] | galactose | Zeaxanthin: 0.01% of CDW  ~0.2 - 0.05 mg/g [CDW]  β-Carotene: 0.103 mg/g [CDW]  Lycopene: 0.113 mg/g [CDW] | [221-223]  [221-223]  [222,223] |
| *Candida utilis* | 1. Introduction of synthetic, codon-optimized *Erwinia uredovora* carotenoid biosynthesis genes (crtE, crtB, crtI and crtY) [216] and *Agrobacterium aurantiacum* carotenoid biosynthesis genes (crtZ and crtW) [217] under the control ot *C. utilis* promoters and terminators:    - Astaxanthin: *crtE*, *crtB*, *crtI*, *crtY*, *crtZ* and *crtW*    - β-Carotene: *crtE*, *crtB*, *crtI* and *crtY*    - Lycopene: *crtE*, *crtB* and *crtI* 2. Improving Lycopene yields [218] by disruption of the *C. utilis* squalene synthase gene (*ERG9*) and overex-pression of the catalytic domain of the *C. utilis* 3-hydroxy methylglutaryl CoA reductase gene (*HMG*) | glucose | Lycopene: 1.1 mg/g [CDW]^[219]^  7.8 mg/g [CDW]^[218]^  β-Carotene: 0.4 mg/g [CDW]  Astaxanthin: 0.4 mg/g [CDW] | [219]  [219]  [218,219] |
| *Saccharomyces cerevisiae* (*fen1*) | 1. Transfer of expression cassettes for mature bovine adrenodoxin (ADX), adrenodoxin reductase (ADR), and side chain cleavage cytochrome P450 (P450scc) 2. Transfer of *Arabidopsis thaliana* Δ7-sterol reductase 3. Disruption of Δ22-sterol desaturase (one step of endogenous ergosterol biosynthetic pathway) | galactose | 60 mg/L | [241] |
| *Saccharomyces cerevisiae* (*fen1*) | 1. Transfer of expression cassettes for mature bovine adrenodoxin (ADX), adrenodoxin reductase (ADR), and side chain cleavage cytochrome P450 (P450scc) 2. Transfer of *Arabidopsis thaliana* Δ7-sterol reductase 3. Disruption of Δ22-sterol desaturase (one step of endogenous ergosterol biosynthetic pathway) 4. Introduction of type II human 3β-hydroxy-steroid dehydrogenase-isomerase (3β-HSD) | galactose | No value given | [241] |
| *Saccharomyces cerevisiae* | 1. Rerouting the ergosterol biosynthesis pathway 2. Introduction of the mammalian-specific part of the hydrocortisone biosynthetic pathway 3. Inactivation of side reactions to steroid biosynthesis dead ends 4. Adjusting expression levels for optimized steroid channeling to hydrocortisone | glucose / ethanol | 11.5 mg/L | [228] |
| *Saccharomyces cerevisiae* | 1. Introduction of the *Artemisia annua* epi-cedrol synthase gene 2. Overexpression of a truncated Hydroxy-methylglutaryl CoA reductase (trHmg1p) 3. Mutation of the Upc2p transcription factor 🡪 introduction of the *upc2-1* allele with G888D [242] 4. Employing the *S.c.* haploid mating type **a** | galactose | 0.37 mg/L | [229] |
| *Saccharomyces cerevisiae* | 1. Introduction of five Taxol biosynthetic genes from *Taxus* species: geranylgeranyl disphosphate syntha-se (GGPPS), taxadiene synthase (TS), taxadiene 5α-hydroxylase (THY5a), taxadienol 5α-*O*-acetyl trans-ferase (TAT), taxoid 10β-hydroxylase (THY10b) with necessary modifications for the expression in *S. c.* 2. Due to restricted THY5a expression, only a very small amount of the intermediate taxadien-5α-ol and no taxadien-5α-acetoxy-10β-ol was detected *in vivo* [230] | simple sugar (glucose, galactose) and [2-^14^C] mevalonic acid for radio-HPLC analysis | taxadien-5α-ol: 0.025 mg/L  no taxadien-5α-acetoxy-10β-ol *in vivo* | [230] |
| *Saccharomyces cerevisiae* | 1. Engineering the farnesyl pyrophosphate (FPP) biosynthetic pathway 2. Introduction of the *Artemisia annua* L amorphadiene synthase gene (FPP 🡪 amorphadiene) 3. Cloning the *A. annua* CYP71AV1/CPR (3-step oxidation: amorphadiene 🡪 artemisinic acid) | simple sugar | ~ 32 mg/L | [226] |
| *Saccharomyces cerevisiae* | 1. Follow-up study of [226]: 2. Engineering the pyruvate dehydrogenase bypass (pyruvate to acetyl-CoA) by overexpression of    - *Salmonella* acetyl-CoA synthetase variant (L641P)    - *S. cerevisiae* cytosolic acetaldehyde dehydrogen-ase (*ALD*6)    - In strain *S. cerevisiae* EPY224 [226]   3) Results: increased levels of mevalonate and amorpha-4,11-diene (~120 mg/L); generally applicable for isoprenoid production | glucose | ~380 mg/L  ~120 mg/L | [227] |
