# Supplementary material for: Yeast cell factories for fine chemical and API production
Source: Microb Cell Fact. 2008 Aug 7;7:25. doi: 10.1186/1475-2859-7-25 (PMC2628649; doi:10.1186/1475-2859-7-25)
Supplement: Additional file 6 — Table 6. [file 1475-2859-7-25-S6.doc]

## Table 6: Synthetic pathways for polyketide and flavonoid synthesis

| **Host organism** | **Engineering steps** | **Substrate** | **Product / Outcome** | **Ref** |
| --- | --- | --- | --- | --- |
| *Saccharomyces cerevisiae* | 1. Introduction of the *Penicillium patulum* PKS 6-MSAS^I^ gene 2. Overexpression of the *Bacillus subtilis* surfactin P-pant^II^ transferase (Sfp) gene | YPD (+ glucose) | 1.7 g/L  (2-fold more than by natural host *Penicillium patulum*) | [232] |
| *Saccharomyces cerevisiae* | 1. Introduction of the *Penicillium patulum* PKS 6-MSAS^I^ gene 2. Introduction of the surfactin P-pant^II^ transferase gene from *Bacillus subtilis* (*sfp*) or from *Aspergillus nidulans* (*npgA*) | galactose minimal medium | >200 mg/L  for strain expressing the PPTase^II^ from *A. nidulans* | [231] |
| *Saccharomyces cerevisiae* BJ5464  (protease deficient) | 1. Introduction of pathways for methylmalonyl-coenzyme A production:   Propionyl-CoA-dependent route:   - - *Salmonella typhimurium* propionyl-CoA synthetase   - *Streptomyces coelicolor* propionyl-CoA carboxylase pathway (PCC)   Propionyl-CoA-independent route   - - *Streptomyces coelicolor* malonyl/methylmalonyl-CoA ligase pathway (MatB)  1. Expression of module 2 from DEBS1^III^ linked to the thioesterase domain (TE) of DEBS3 [243,244] 2. Co-expression of five tRNA genes E4, R2, L5, Q2 and P2 [245] | YPD (+ glucose)  + propionate and propyl-diketide thioester feed | 0.5-1 mg/L  with propionyl-CoA-dependent route (PCC-pathway) | [215] |
| *Saccharomyces cerevisiae* | 1. Introduction of the *Populus trichocarpa* X *Populus deltoides* (= poplar) cDNAs encoding:    - Phenylalanine ammonia lyase (isoform PAL2 and PAL4, respectively)    - Cinnamate 4-hydroxylase (C4H)    - Cytochrome P450 reductase (CPR2) | selective, synthetic medium supplemented with glucose and galactose, respectively  feed with [^3^H]phenylalanine  [^14^C]cinnamate | ~ 3-10 mg/L  triple-expressing strains (PAL2/C4H/CPR2 and PAL4/C4H/CPR2, respectively; with slight advantages for the PAL2 expressing strain) | [233] |
| *Saccharomyces cerevisiae* | 1. Introduction of the *Helianthus tuberosus* C4H Cytochrome P-450 and *H. tuberosus* NADPH-Cytochrome P-450 reductase 2. Introduction of the *Rhodotorula glutinis* ATCC 10788 phenylalanine ammonia-lyase (PAL) | glucose or raffinose  addition of L-phenylalanine (1.0 mM)  induction with galactose | after 24 h:  on glucose: 354 µM  (🡪 58 mg/L)  on raffinose: 498 µM  (🡪 82 mg/L) | [234] |
| *Saccharomyces cerevisiae* AH22 | 1. Introduction of the phenylpropanoid pathway:    - *Rhodosporidium toruloides* phenylalanine ammonia lyase (*PAL*)    - *Arabidopsis thaliana* 4-coumarate:coenzyme A (CoA) ligase (*4CL*)    - *Hypericum androsaemum* chalcone synthase (*CHS*) | YPD (+ glucose) and  YPL (+ galactose) | *S. cerevisiae* AH22 with PAL, 4CL and CHS produced ~ 7 mg/L of naringenin and 0.8 mg/L of pinocembrin | [235] |
| *Saccharomyces cerevisiae* YPH499 | 1. Introduction of the chalcone synthase (CHS) from ripe raspberry (*Rubus idaeus*) or a variant thereof (CHS L214I-F215L) 2. Introduction of the tobacco 4-coumarate-coenzyme A ligase (4CL) | YPGal-medium  (induction with galactose)  addition of *p*-coumaric acid (3 mM) | raspberry fruit:  1-4 mg/kg  recombinant  *E. coli*: 5 mg/L    recombinant *S. cerevisiae*: no proper detection of raspberry ketone | [237]  [236]  [236] |
| *Saccharomyces cerevisiae* FY23 | 1. Introduction of the coenzyme-A ligase *4CL216* gene from hybrid poplar under the control of the yeast ADH2 gene promoter and terminator (🡪 CAL1) and 2. the resveratrol synthase (VST1) from grapevine (*Vitis vinifera*) | SCDL-medium (0.67% yeast nitrogen base, 0.8% glucose and required growth factors)  with 10 mg/L *p*-coumaric acid | recombinant  *S. cerevisiae*:  ~1.5 µg/L | [238] |
| *Saccharomyces cerevisiae*  CEN.PK 113-3b  (*ura3 his3*) | 1. Introduction of the 4-coumarate: coenzyme A (CoA) ligase (*4CL2* gene – GenBank accession no. U50846) from *Nicotiana tabacum* cv. Samsun 2. Introduction of the *STS* gene from *Vitis vinifera* encoding for the stilbene synthase (STS) | 50 mL yeast nitrogen base medium  supplemented with 5 mM *p*-coumaric acid and 2% galactose to induce gene expression | *S. cerevisiae*:  ~6 mg/L  recomb. *E. coli* expressing the same enzymes:  ~16 mg/L | [239] |

^I^PKS = polyketide synthase; 6-MSAS = 6-methylsalicylic acid synthase

^II^PPTase = P-pant transferase = 4’-phophopantetheinyl transferase

^III^DEBS = deoxyerythronolide B synthase from *Saccharopolyspora erythraea*, a typical ‘modular’ polyketide synthase.
